# PhiCube: a reconfigurable bilateral robotic device for neurorehabilitation

**DOI:** 10.3389/fmed.2026.1829129

**Published:** 2026-07-13

**Authors:** Matteo Lavit Nicora, Giovanni Tauro, Atul Chaudhary, Davide Felice Redaelli, Matteo Malosio

**Affiliations:** 1 Institute of Intelligent Industrial Technologies and Systems for Advanced Manufacturing, National Research Council, Lecco, Italy; 2 Rehabilia Technologies Srl, Milano, Italy; 3 Industrial Engineering Department, University of Bologna, Bologna, Italy

**Keywords:** bilateral upper-limb rehabilitation, modular kinematic architecture, pediatric neuromotor disorders, play-based motor learning, robotic rehabilitation device

## Abstract

**Introduction:**

Neuromotor disorders affecting upper-limb function represent a substantial clinical challenge in pediatric populations, with conditions such as hemiplegic cerebral palsy imposing lasting limitations on functional independence and quality of life. Bilateral motor training has emerged as a neurophysiologically grounded paradigm, offering functional advantages over purely unilateral approaches by actively exploiting interlimb coordination mechanisms. Despite the growing evidence base for bilateral training, existing robotic devices present critical limitations in kinematic reconfigurability, parameterizable inter-limb coupling, and portability, restricting their clinical accessibility and therapeutic versatility, particularly in pediatric settings.

**Methods:**

This paper introduces PhiCube, a modular robotic platform engineered for bilateral upper-limb neuromotor rehabilitation in pediatric populations. The device architecture is built around a compact central body housing two independent motorized rotational joints, whose actuation axes can be rapidly aligned with any of the three principal anatomical planes through a dedicated reconfiguration mechanism. A set of interchangeable therapeutic manipulanda, each eliciting distinct motor patterns, extends the device's therapeutic scope across the proximal-to-distal upper-limb kinematic chain. The bilateral coupling controller is formulated as an impedance-based scheme exposing tuneable parameters governing the kinematic transmission ratio between limbs, the directional asymmetry of the coupling, and the bilateral assistance level. A gamification environment embeds motor training within interactive paradigms designed for pediatric engagement, with real-time mapping between game trajectories and robotic assistance references. Kinematic simulations were conducted within the OpenSim framework, integrating an upper-extremity musculoskeletal model through a custom Python-based inverse kinematics pipeline.

**Results:**

Kinematic simulations confirmed that each handle–orientation combination elicits a distinct and characterizable pattern of joint recruitment across the upper-limb kinematic chain, with configurations ranging from isolated distal training (wrist flexion–extension, forearm pronation–supination) to compound multi-joint synergies engaging the shoulder, elbow, and wrist simultaneously. Analysis of the bilateral controller through contour mapping of the torque field demonstrated that the coupling parameters shape the inter-limb interaction, spanning configurations from unilateral guidance—in which one limb drives the other without reciprocal constraint—to fully symmetric bilateral coupling, and from in-phase synchronous to counter-phase alternating coordination.

**Discussion:**

The parametric characterization of both the kinematic architecture and the control space provides objective evidence that PhiCube can address a broad and clinically relevant spectrum of bilateral rehabilitation paradigms, from proximal multi-joint synergies to isolated distal training, and from strict guided-movement to symmetric and counter-phase bilateral coordination. These properties position PhiCube as a versatile tool for neuromotor rehabilitation, where therapeutic goals, residual motor capacity, and anthropometric constraints vary substantially across patients and evolve throughout the course of treatment.

## 1 Introduction

### 1.1 Background and motivation

Neuromotor disorders affecting upper limb function represent a substantial clinical challenge in pediatric populations worldwide. These conditions encompass a heterogeneous group of disabilities that impair the normal execution of motor gestures, significantly affecting children's capacity to perform activities of daily living, participate in educational and social contexts, and achieve developmental milestones. Among pediatric neuromotor disorders, cerebral palsy (CP) stands as the most prevalent motor disability in childhood, with global estimates indicating an incidence of approximately 2 to 3 cases per 1,000 live births (1). Cerebral palsy serves as a paradigmatic condition for understanding the challenges of pediatric upper limb rehabilitation: it frequently presents with asymmetric motor involvement (as in hemiplegic forms), affects multiple joints simultaneously, and requires long-term therapeutic intervention throughout the developmental period. Notably, the majority of children with CP exhibit impairment of upper extremity function, which profoundly impacts their functional independence and quality of life (2).

The consequences of upper limb neuromotor impairments in children extend far beyond physical limitations. These conditions affect psychosocial development, school participation, and overall quality of life for both patients and their caregivers (3). Early and intensive intervention is therefore critical, as the developing nervous system exhibits heightened neuroplasticity, offering a unique window of opportunity for functional recovery through appropriate therapeutic stimulation (4). This neuroplastic potential underscores the importance of providing intensive and engaging rehabilitation during childhood.

Traditional rehabilitation approaches for upper limb dysfunction typically rely on repetitive, task-oriented exercises delivered by physical and occupational therapists. While effective, these conventional methods present significant challenges: they are highly dependent on therapist availability, physically demanding for clinical staff, and often struggle to maintain the engagement and motivation of young patients over extended treatment periods. The rehabilitation process is inherently characterized by high repetitiveness and prolonged duration, representing a considerable challenge for both patients and healthcare professionals involved in the therapeutic journey.

A fundamental consideration in pediatric rehabilitation is the role of play as a vehicle for learning and adaptation (5). Unlike adults, children naturally learn and develop skills through play; this is not merely a form of entertainment but rather a primary mechanism through which children explore their environment, acquire new abilities, and adapt to challenges. This principle is foundational in pediatric rehabilitation: therapeutic interventions that incorporate play-based elements can support children's developmental needs while simultaneously addressing motor deficits. Play provides a dynamic and engaging context that facilitates learning and promotes adaptability, making it an essential component of effective pediatric rehabilitation strategies.

Robotic rehabilitation devices have emerged as promising tools to complement traditional approaches. These systems offer distinct advantages including precise movement control, consistent repetition without therapist fatigue, objective performance measurement through integrated sensors, and the capacity to deliver adaptive assistance based on patient capabilities (6, 7). The sensors integrated into robotic devices enable constant monitoring of motor capabilities, providing quantitative data that can inform clinical decision-making and track progress over time. Furthermore, robotic platforms can incorporate interactive software interfaces to enhance motivation and treatment adherence.

A particularly relevant paradigm in neuromotor rehabilitation is bilateral arm training, which involves simultaneous engagement of both upper limbs during therapeutic exercises. This approach is grounded in neurophysiological evidence demonstrating that bilateral movements facilitate enhanced activation of the primary motor cortex (8) and promote interhemispheric communication (9). The premise underlying bilateral rehabilitation is that, during simultaneous movements, the less-affected upper limb can support the movement of the more-affected limb at the cerebral level. For conditions characterized by asymmetric motor impairment—such as hemiplegic cerebral palsy or other forms of hemiparesis—bilateral training enables functional coupling between limbs, leveraging interlimb coordination to drive motor learning and cortical reorganization (10). Systematic reviews have demonstrated that bilateral training can be more effective than unilateral approaches in terms of motor recovery, with evidence supporting its application (11, 12).

However, the development of bilateral robotic devices specifically designed for pediatric populations, remains limited. The field of medical device development has historically prioritized adult applications, resulting in a pediatric landscape populated largely by adapted adult devices rather than purpose-built solutions. This approach fails to adequately account for critical differences in children, including anthropometric variability across developmental stages, distinct cognitive and attentional profiles, different disease presentations, and the fundamental need for engagement through play. Consequently, there exists a compelling need for rehabilitation robots conceived from inception to address the unique physiological, developmental, and engagement requirements of pediatric users.

### 1.2 State of the art

The field of robotic upper limb rehabilitation has witnessed substantial growth over the past two decades, yielding diverse technological solutions spanning passive unactuated devices, end-effector robots, and exoskeletal systems (6, 7). However, when evaluated through the lens of pediatric bilateral rehabilitation requirements, significant technical limitations become apparent across the existing technological landscape. The following sections provide a critical analysis of current approaches, highlighting the gaps that motivate the development of novel solutions.

#### 1.2.1 Passive devices

Among the earliest approaches to bilateral upper limb training are passive, non-motorized systems that mechanically couple the movements of both arms. The Bilateral Arm Training with Rhythmic Auditory Cueing (BATRAC) system represents a foundational device in this category, consisting of two independent T-bar handles mounted on low-friction tracks that permit forward-backward pushing and pulling movements (13). While BATRAC demonstrated therapeutic efficacy in post-stroke adult populations, it presents several limitations: its mechanical configuration restricts motion to a single plane, it is not reconfigurable for different movement patterns, and it focuses primarily on proximal joints (shoulder and elbow) without addressing hand and wrist function. A modified version was later developed to address distal upper limb function (14), but this required a separate device configuration rather than a unified reconfigurable platform.

Similar limitations characterize the Active-Passive Bimanual Therapy device (APBT, also known as “The Rocker”), which provides a mechanism for bilateral wrist flexion-extension training through coupled crankshafts (15). While supporting mirror-symmetrical coordination of wrist movements, this device constrains motion to the horizontal plane and does not permit independent orientation of movement directions. The involvement of proximal upper limb joints (shoulder and elbow) is not addressed.

The Reha-Slide system family offers additional passive bilateral options. The Reha-Slide Duo features two sledges on parallel tracks that can move independently, while the Reha-Slide variant adds a connecting rod between handles, enabling coordinated movement patterns including elbow and shoulder extension-flexion, and wrist rotation (16, 17). However, these systems do not allow independent orientation of the movement guide directions, and the mechanical coupling between handles limits the flexibility of training paradigms.

A common limitation across all passive bilateral devices is the inherent lack of active assistance or resistance capabilities. This restricts their applicability to patients with sufficient residual motor function to generate movement independently, excluding more severely affected individuals who require external assistance to complete therapeutic tasks. Furthermore, none of these systems offer sufficient reconfigurability to address the variety of motor gestures required for comprehensive upper limb rehabilitation.

#### 1.2.2 Robotic devices

Motorized bilateral rehabilitation systems introduce the capability for active assistance and resistance, enabling treatment of patients across a broader spectrum of impairment severity. However, many of these devices present constraints that limit their clinical versatility.

The Bi-Manu-Track system provides robot-assisted bilateral forearm and wrist movements for hemiparetic patients through practice of pronation-supination and flexion-extension (18). Despite its motorized capability, movement is constrained along fixed linear directions, limiting the variety of achievable motor gestures. The device does not permit reconfiguration to address different movement patterns or joint combinations.

The Robot-aided Bilateral Force-Induced Isokinetic Arm Movement Training (BFIAMT) system similarly focuses on bilateral isokinetic movements but maintains directional constraints designed for movements along fixed linear paths (19). This lack of flexibility restricts the range of motor tasks that can be trained, potentially limiting functional transfer to activities of daily living.

The ARCMIME device offers somewhat greater movement flexibility through its articulated structure (20). However, this increased range of motion comes at the cost of a significantly larger physical footprint, limiting its deployment to specialized clinical settings and precluding home-based use. The Bimanual Lifting Rehabilitator provides elevation control based on bilateral hand forces, amplifying patient effort through its actuation system (21). Like other devices in this category, it does not permit appropriate reconfiguration for use with different motor gestures, limiting therapeutic variety.

Some robotic platforms have incorporated accessories to enable bilateral training. The Braccio di Ferro haptic workstation can accommodate bilateral exercises but constrains motion to planar movements without independent limb control (22). This limitation prevents training of the diverse three-dimensional movement patterns required for functional upper limb use. Bimanual handlebar configurations with two degrees of freedom (DOFs) have been developed for bilateral reaching movements, employing velocity-dependent force fields to study motor adaptation (23). However, such systems constrain movement to the sagittal plane with focus on shoulder and elbow joints, and do not offer kinematic reconfigurability for training diverse motor gestures across multiple joint combinations.

The Driver's SEAT (Simulation for Arm Therapy) system implements a car-steering paradigm for bilateral upper limb rehabilitation, demonstrating creative application of functional task training (24). However, the single rotational degree of freedom and fixed steering-wheel configuration constrain the system to a specific movement pattern, limiting its applicability across the range of motor gestures required for comprehensive upper limb rehabilitation.

At the highest complexity level, exoskeletons offer comprehensive multi-joint capabilities covering shoulder, elbow, wrist and hand, enabling training across a wide range of motion and functional tasks (25, 26). Among these, some exoskeletons have been designed with a bilateral configuration to enable mirrored training of both upper limbs (27). However, high costs and considerable physical bulk confine these systems to specialized clinical environments, while lengthy setup and configuration procedures further reduce the number of sessions that can be realistically delivered. Taken together, these constraints limit accessibility and prevent sufficiently intensive treatment for a large number of children.

#### 1.2.3 Devices addressing pediatric rehabilitation

Within the broader landscape of upper limb rehabilitation robotics, relatively few technological solutions have been specifically developed for pediatric populations. A comprehensive review of robotic devices for pediatric rehabilitation identified that the majority of existing systems were originally designed for adult patients, with pediatric applications relying on subsequent adaptations rather than purpose-built engineering (28). This design approach presents inherent limitations, as children differ substantially from adults in anthropometric dimensions, biomechanical properties, cognitive development, and engagement requirements. Systematic reviews examining robot-assisted therapy for upper limb function in children with cerebral palsy have reported positive effects on motor function, but identified a limited number of robotic systems actually employed in pediatric clinical studies (29).

Among exoskeletal approaches, the Armeo^®^Spring Pediatric represents an exoskeleton-like system providing antigravity support providing three-dimensional workspace coverage through passive mechanisms. However, the absence of active actuation limits the capacity for real-time adaptive assistance modulation and precludes resistance-based training protocols. The Diego^®^ platform is a ceiling-mounted antigravity platforms offering three-dimensional movement capability but provides assistance exclusively in the vertical direction, constraining the range of trainable functional movements and limiting deployment flexibility due to infrastructure requirements. The ChARMin exoskeleton represents one of the few actuated systems specifically designed for children, featuring six DOFs for shoulder, elbow, forearm, and wrist movements, with adaptability to patients aged 5 to 18 years (30). Notwithstanding their broad kinematic coverage, exoskeletal systems share a set of limitations that hinder their widespread clinical adoption: considerable physical bulk and weight restrict deployment to specialized environments, time-consuming setup and fitting procedures may reduce patient comfort and preclude rapid deployment across diverse clinical settings, and high acquisition and maintenance costs confine their use to centers with substantial dedicated budgets.

End-effector robotic systems have been employed in pediatric populations both for therapeutic intervention (31) and as quantitative assessment tools, demonstrating good correlation between robot-derived kinematic measures and clinical scales (32). However, the majority of platforms used in pediatric rehabilitation are derived from adult-oriented designs, rather than being engineered from inception to meet the distinctive developmental, anthropometric, and engagement requirements of children (33). Critically, while the neurophysiological rationale for bilateral training is well established, its robotic implementation remains underdeveloped: fixed mechanical coupling between limbs precludes the parameterizable inter-limb relationships necessary to systematically exploit interlimb coordination mechanisms across the spectrum of clinical presentations.

Design requirements specific to pediatric rehabilitation robots include adaptability to continuous developmental changes, minimized device weight to avoid hindering movement, robust safety mechanisms accounting for children's limited hazard assessment capabilities, and motivation-enhancing features such as engaging interfaces and game-based interactions (28). Current technological solutions address these requirements with varying degrees of success, with significant gaps remaining in the integration of bilateral training capabilities, kinematic reconfigurability, and portability within pediatric-specific platforms.

#### 1.2.4 Identified gaps in current technologies

Critical analysis of the state of the art reveals technological gaps that limit the effectiveness and deployability of current rehabilitation systems for bilateral upper limb training:

1. Kinematic reconfigurability: Most bilateral devices constrain movement to fixed directions or planes, restricting the variety of motor gestures that can be trained. The possibility of executing rehabilitative movements linked to different motor patterns through a single reconfigurable platform instead of multiple specific solutions requiring extensive economic resources and personnel training, remains largely unaddressed in current technological solutions.
2. Bilateral control architecture: Many devices providing bilateral capability do not permit truly independent limb control or flexible, parameterizable coupling relationships between limbs. Effective bilateral rehabilitation requires control architectures capable of establishing adjustable functional relationships between limb movements to exploit interlimb coordination mechanisms.
3. Portability and deployment accessibility: Complex robotic systems typically require dedicated clinical infrastructure, creating barriers to home-based or community-based rehabilitation that could enhance treatment intensity and continuity—factors known to influence neuroplastic recovery.

A further limitation concerns the inadequate integration of play-based motor learning: few systems treat play as a fundamental mechanism for skill acquisition rather than a mere engagement feature, leaving the neurophysiological principles underlying play-based learning largely underexploited. Finally, most rehabilitation robots target adult populations, with pediatric applications relying on scaled-down adaptations rather than systems engineered from inception to address children's unique anatomical and biomechanical characteristics.

### 1.3 Contribution and objectives

In response to the identified technological limitations, this paper introduces PhiCube, a modular robotic platform engineered for bilateral upper limb rehabilitation in pediatric populations. By leveraging interlimb coordination mechanisms through its reconfigurable architecture, PhiCube facilitates diverse bilateral motor gestures, providing a significant functional advantage over solely unilateral systems within a compact, versatile form factor. This pediatric-centered device integrates play-based motor learning with multimodal sensory feedback, ensuring therapeutic engagement is intrinsically driven by interactive paradigms. Furthermore, its control architecture allows for continuous assistance-resistance modulation and parameterizable bilateral coupling, while embedded sensors automate performance quantification to provide objective, data-driven insights for precise clinical monitoring and treatment optimization.

This article describes the mechanical concept, derives the kinematic model, and presents the bilateral control system that facilitates coordinated movement between the two arms. Kinematic simulations demonstrate the platform's capacity to facilitate diverse upper limb motor patterns. By leveraging reconfigurable architectures and interchangeable manipulanda, the device can selectively target specific joint assemblies, tailoring kinematic engagement to meet various therapeutic requirements.

## 2 Materials and methods

### 2.1 PhiCube

**Figure 1:**
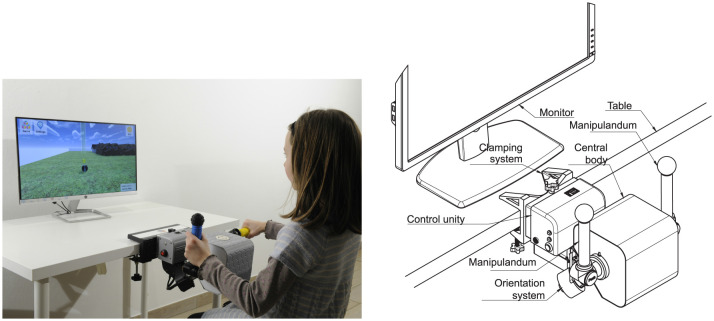
Overall view of PhiCube. The device comprises the central body with two motorized joints, the base clamping unit, and the control electronics. A monitor is positioned on the table surface to display the gamification interface during therapy sessions.

PhiCube is a rehabilitation robotic device designed around a modular architecture that enables bilateral upper limb training through kinematic reconfigurability and adaptable end-effector configurations. Figure 1 shows a general view of the device, connected to a table on which a monitor is positioned. The device is composed of the following main components:

- Central body—Motorized and sensor-equipped unit, to which the Manipulanda for performing the exercises are connected.
- Connection unit—Houses PhiCube's electrical and electronic components, and provides the electrical/electronic connections between the device and the external PC.
- Orientation system—Mechanical subsystem connecting the central body to the clamping unit, enabling rapid reconfiguration of the actuation axes across the three principal anatomical planes.
- Clamping system—Mechanical component that can be clamped to a table and connected to the rest of the system through the dedicated sliding interface.
- Manipulanda—Hand-held interchangeable accessories through which patient interaction occurs. Different types of manipulanda allow different motor gestures.

As detailed in Section 2.1.1, the central robotic body is equipped with two motorized rotational axes featuring a quick-attachment mechanical interface that enables rapid connection and interchange of purpose-designed manipulanda.

The actuation system can operate in multiple modes depending on therapeutic requirements and patient capabilities (see Section 2.1.3). Each axis can function independently as: a passive measurement system for assessment, a movement actuator providing active assistance, an amplifier of the force exerted by the patient, or a resistive element providing therapeutic challenge. In bilateral mode, the control system establishes functional relationships between the movements of both limbs, with adjustable transmission and assistance parameters. This enables therapeutic paradigms where the less-impaired limb guides the more-impaired limb, with the degree of coupling and assistance individually tailored to each patient's needs and progression.

A dedicated gamification environment (Section 2.1.4) embeds motor and cognitive training within interactive game-based scenarios, providing real-time mapping between game trajectories and robotic assistance references to sustain engagement and motivation throughout the rehabilitation session.

Moreover, the device integrates a comprehensive software platform for therapy session management and a dashboard for performance monitoring. All usage data are automatically recorded and processed to generate objective performance indices, facilitating evidence-based tracking of patient progress.

Its lightweight construction, modular architecture, and tool-free table-mounting interface make PhiCube easily transportable and rapidly deployable across hospital, outpatient, and home-based settings, supporting treatment intensity and continuity beyond the clinical environment.

The device is currently protected by an industrial invention patent (34) and has served as an experimental platform in multimodal rehabilitation studies, where its capacity to deliver adjustable assistance—including in bilateral mode—was exploited to investigate combined robotic and non-robotic interventions (35).

#### 2.1.1 The central body

The central body houses the actuation and sensing components within a compact enclosure equipped with two independent motorized rotary joints. Each joint is instrumented to enable real-time measurement of joint kinematics and interaction forces.

The rotary axes terminate in quick-coupling mechanical interfaces that facilitate rapid interchange of therapeutic manipulanda without tools. The coupling system incorporates an angular reference that ensures handle connection in a uniquely defined configuration with respect to the actuated joint. The coupling interface design accommodates different manipulanda, expanding the workspace geometry to support diverse rehabilitation protocols (Section 2.1.2).

**Figure 2:**
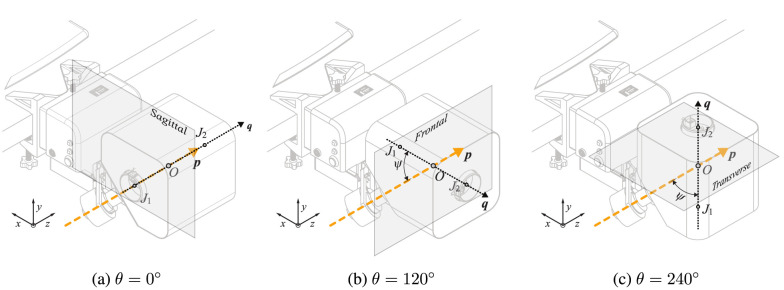
The three discrete orientations of the PhiCube central body, obtained by rotating the central body about the reconfiguration axis *p* in increments of 120°. The central body houses two motorized joints *J*_1_ and *J*_2_ mounted on opposite faces; the axis *q*, directed from *J*_1_ to *J*_2_ and coinciding with the common rotation axis of both actuators, defines the direction along which motor-driven motion is imparted to the attached manipulanda. Positive rotation about *q* is defined following the right-hand rule about *q*. The axis *p*, fixed to the clamping system, forms an angle $\psi =\arctan\left(\sqrt{2}\right)\approx 54.74^{˙}$ with respect to *q*, enabling the three orientations to align *q* perpendicularly to the sagittal **(a)**, frontal **(b)**, and transverse **(c)** anatomical planes, respectively. The directions of the reference frame axes are defined with respect to the standard operating posture of the subject relative to the device, and is consistent with the model employed in the OpenSim simulations (Section 2.2). **(a)** θ = 0°. **(b)** θ = 120°. **(c)** θ = 240°.

**Figure 3:**
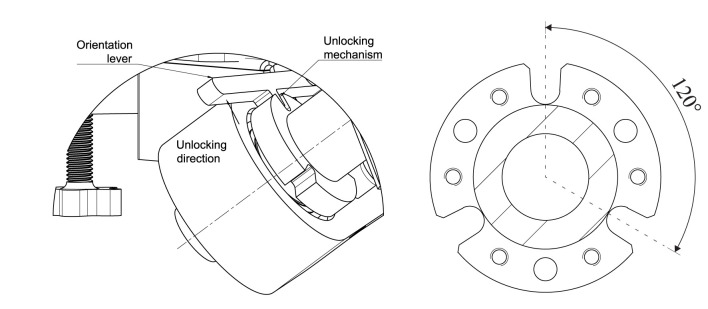
Orientation locking lever. When the user moves the lever in the unlocking direction **(left)**, the mechanical tooth disengages and allows the rotation of the reconfiguration system. As the next discrete rotation step is reached **(right)**, a spring mechanism pulls the lever back locking the system.

A distinctive feature of the PhiCube architecture is the kinematic reconfiguration system that connects the central body to the base unit. This connection employs a rotary joint with a strategically oriented axis (Figure 2). The axis of this reconfiguration joint is positioned at an angle of $\psi =\arctan\left(\sqrt{2}\right)\approx 54.74^{˙}$ relative to the actuated motor axes. This specific angular configuration enables the motor axes to be reoriented perpendicular to each of the three principal anatomical planes: sagittal, frontal, and transverse. Geometrically, this is equivalent to rotating a cube about its space diagonal, which cyclically permutes the cube's faces with respect to the three anatomical planes. By rotating the central body around the inclined axis, the system can sequentially align the actuation axes normal to any of these orthogonal planes, thereby providing three distinct kinematic configurations while maintaining the center of the cube in the same position. A mechanical lever maintains the selected orientation by locking the rotation of the inclined axis at three discrete positions spaced 120° apart, corresponding to the three anatomical plane orientations. To reconfigure the device orientation, the user lifts the unlocking lever, rotates the central body to the desired position, and releases the lever to engage the locking mechanism (Figure 3).

#### 2.1.2 The manipulanda

**Figure 4:**
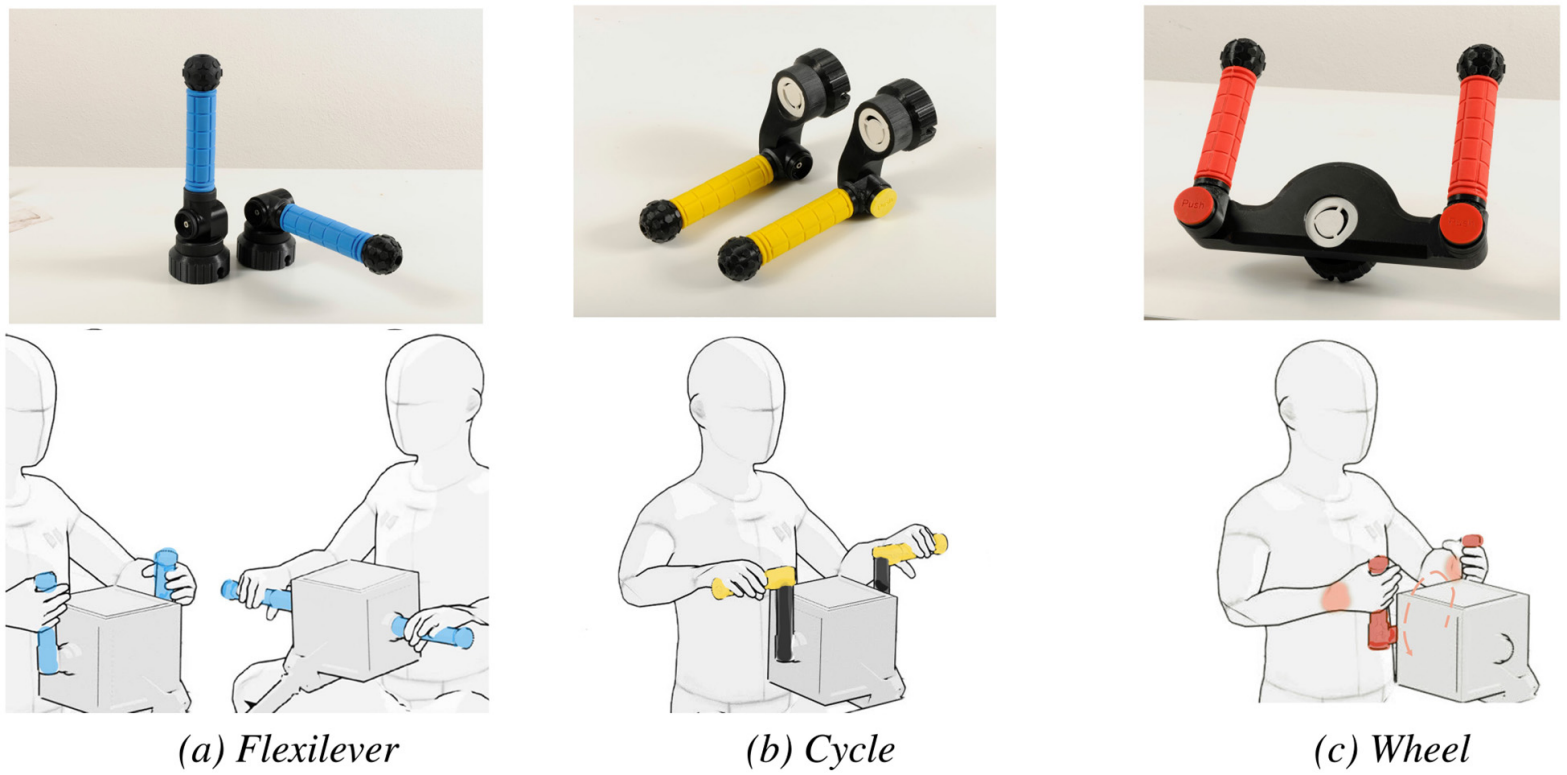
On the left the *FlexiLever* manipulanda allowing either isolated wrist movements or compound shoulder-elbow patterns. In the center the *Cycle* manipulanda used for rhythmic rotary movements. On the right the *Wheel* manipulandum simulating a steering wheel or a bicycle handlebar. **(a)** Flexilever. **(b)** Cycle. **(c)** Wheel.

**Figure 5:**
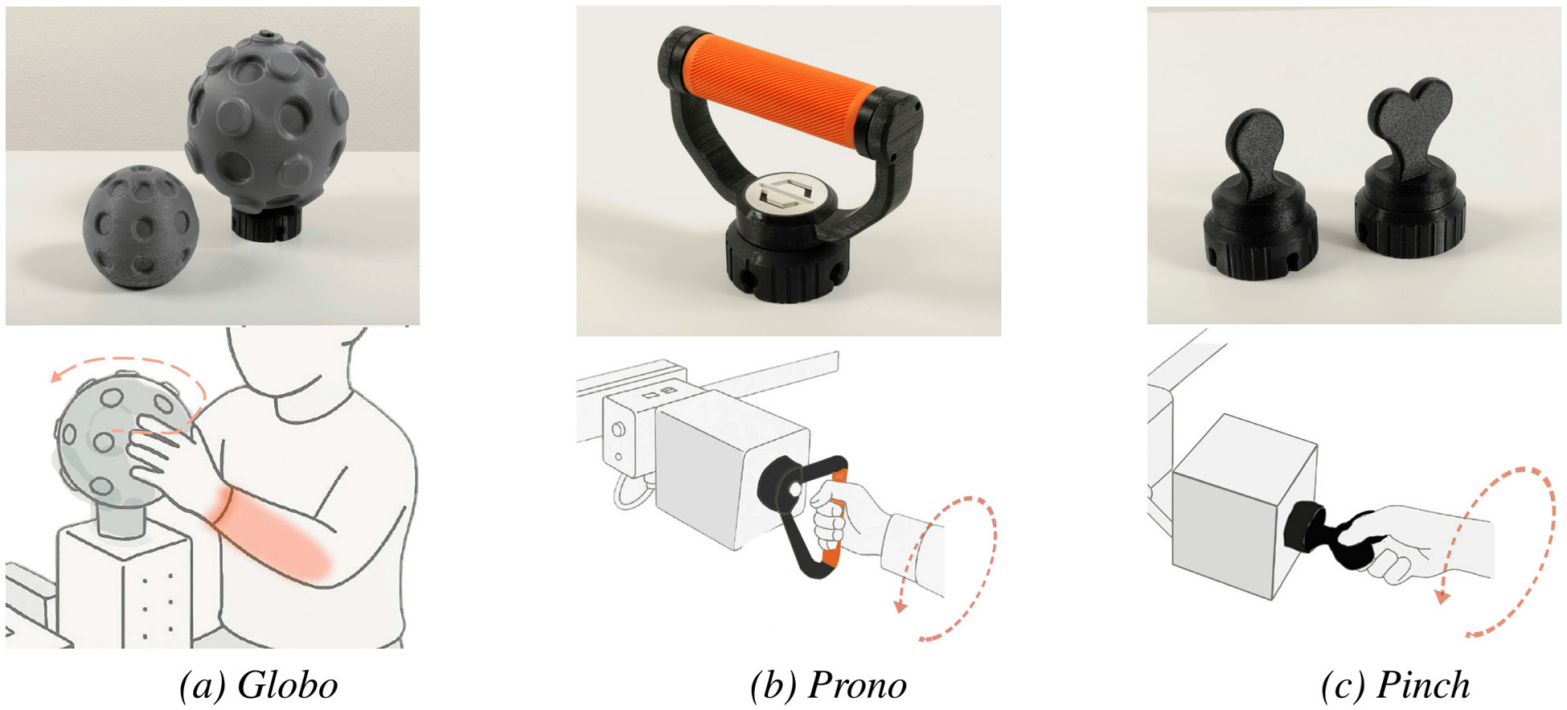
On the left the *Globo* manipulandum designed to simulate manipulation of spherical objects. In the center the *Prono* manipulandum promoting forearm pronation-supination training. On the right the *Pinch* manipulandum targeting fine motor control and digit coordination. **(a)** Globo. **(b)** Prono. **(c)** Pinch.

The device is equipped with a set of manipulanda designed to elicit various upper limb motor patterns through different gripping configurations (Figures 4, 5). Each manipulandum attaches to the central body through the standardized quick-coupling interface, enabling therapists to modify exercise configurations rapidly between or during therapy sessions without tools. The grip surface can be either mechanically constrained or free to rotate about its axis, depending on the handle design and the intended movement pattern.

Therapeutic application is determined by the combination of manipulandum selection and central body orientation, allowing the same device to address diverse rehabilitation objectives through systematic reconfiguration. Optional velcro stabilization straps can be used to assist patients with reduced grip strength or hand function, ensuring consistent mechanical coupling during exercise execution. The system modularity further allows a single manipulandum to be used across multiple central body orientations, thereby expanding the therapeutic scope of each one of them. The following subsections describe the manipulanda currently available for PhiCube.

The *FlexiLever* manipulandum (Figure 4a) provides configurational flexibility through adjustable grip angle settings. A quick release mechanism permits rapid inclination adjustment, enabling progressive training from isolated wrist movements to compound shoulder-elbow patterns. Sagittal plane orientation of the central body (Figure 2a) supports bilateral exercise protocols, while frontal (Figure 2b) or transverse (Figure 2c) orientations enable unilateral training of functional movement patterns, such as door handle operation.

The *Cycle* manipulandum (Figure 4b) is designed to train rhythmic rotary motion patterns, engaging reciprocal coordination patterns. In sagittal plane orientation, bilateral cycling exercises are executed with synchronous or asynchronous phase relationships between limbs, training interlimb coordination and rhythm generation. Frontal or transverse plane orientations permit unilateral circular trajectory training.

The *Wheel* manipulandum (Figure 4c) emulates the biomechanics of automotive steering, simulating a functionally relevant motor task. In frontal plane orientation, the circular geometry with bilateral grips reproduces the coordinated shoulder-elbow synergies required for vehicle operation. Transverse plane orientation alternatively simulates bicycle handlebar control. Adjustable grip orientation is achieved through a quick release mechanism, enabling customization of hand posture to accommodate individual anatomical constraints.

The *Globo* manipulandum (Figure 5a) is available in two diameter variants, both designed to simulate the manipulation of spherical objects and promote palmar opening during interaction. The large-diameter variant is intended for bimanual tasks in which both hands simultaneously engage a single spherical interface, and is optimally deployed with the central body oriented in the frontal or vertical configuration. The small-diameter variant accommodates single-hand grasping and is particularly suited for bilateral exercises in the sagittal configuration, where each limb operates an independent handle.

The *Prono* manipulandum (Figure 5b) is engineered to promote forearm pronation-supination training while distributing contact forces across the palmar surface. This manipulandum is designed to be deployed in unilateral mode with the central body oriented in the frontal plane, promoting forearm rotation while minimizing compensatory shoulder or trunk movements.

The *Pinch* manipulandum (Figure 5c) combines forearm pronation-supination with precision grip training, targeting fine motor control and digit coordination. Two variants address different therapeutic objectives: the “butterfly” configuration provides an extended contact surface for the index finger, facilitating ergonomic pinch postures that reduce digit fatigue during repetitive exercises. The “key” configuration presents a reduced contact area requiring higher pinch forces, thereby increasing demand on intrinsic hand musculature. Both variants are primarily utilized in unilateral configuration with frontal plane orientation, enabling training of precision grip patterns relevant to activities of daily living.

#### 2.1.3 Control system

The algorithm responsible for tuning and controlling the behavior of the two motorized axes has been designed to accommodate and leverage the intrinsic flexibility and adaptability of the device. As presented, PhiCube is capable of offering monolateral, independent bilateral and constrained bilateral exercise paradigms which can all be realized through a properly tuned and sufficiently flexible robotic controller. The control of each motorized axis is realized through an impedance-control scheme with torque as the commanded variable. At every control cycle, the algorithm reads the instantaneous angular positions of both joints from the encoders, evaluates the command torque τ_*i*_, and forwards it to the current/torque loop of each motor driver. With this approach, the controller shapes the mechanical impedance perceived at the handles without imposing any kinematic constraint on the patient. First of all, it is important to distinguish between two therapeutic cases. The first case can be referred to as *decoupled training*, when either only one of the axes is used to realize a specific monolateral exercise or both of them are activated but behave independently from each other. The second case is called *coupled training*, when the two axes are activated simultaneously, and their behavior is driven by a specific law that constrains their reciprocal movement.

Now, the torque to be generated by the control algorithm for each joint *i*∈{1, 2} can be defined as the sum of two contributions in Equation 1:


$$\begin{array}{l} \tau_{i}=\tau_{i,a}+\tau_{i,b} \end{array}$$ (1)

where τ_*i, a*_ is the *reference component* and τ_*i, b*_ is the *bilateral component*. Both contributions are commanded as torques of each motor driver; the expressions reported below define virtual visco-elastic fields acting on the joint. Depending on the logic (coupled or decoupled) chosen for the specific application, these two torque components are computed in different ways, as hereafter.

##### 2.1.3.1 Reference component (τ_*i, a*_)

The reference component is used to shape the visco-elastic behavior of each joint around a prescribed angular anchor. Its definition changes between the coupled and decoupled logics.

- *Decoupled logic*. Considering monolateral or decoupled bilateral applications, the reference component depends on four parameters which can be set independently for the two motorized axes: for each *i* joint (a) a gain *k*_*i, a*_ is used to scale the torque magnitude generated in order to define the elastic action around (b) an equilibrium angle θ_*i, a*_, while (c) a second gain *d*_*i, a*_ defines the viscous component related to (d) an equilibrium velocity ${\dot{\theta }}_{i,a}$. With the simple implementation reported in Equation 2, the reference component can be leveraged both for resistive and assistive exercises. If, for instance, θ_*i, a*_ and ${\dot{\theta }}_{i,a}$ are set to zero and the patient is asked to move the handle/s, a resistive visco-elastic force proportional to *k*_*i, a*_ and *d*_*i, a*_ will be generated. If, on the contrary, the equilibrium angle and its rate of change are updated along a prescribed low of motion, a force proportional to the chosen gains will be generated to assist the patient in the execution of that specific movement.

$$\begin{array}{l} \tau_{i,a}=k_{i,a}\left(\theta_{i,a}-\theta_{i}\right)+d_{i,a}\left({\dot{\theta }}_{i,a}-{\dot{\theta }}_{i}\right) \end{array}$$ (2)

- *Coupled logic*. Moving to constrained bilateral exercises, the only difference in the definition of the reference component lies in the kinematic constraint imposed to the equilibrium angles θ_*i, a*_ of the two joints. For this purpose, a *transmission ratio* ρ is used to define the consistency condition reported in Equation 3. Non-unity values of ρ accommodate asymmetric ranges of motion between the two sides, while a negative value of ρ inverts the consistent direction, associating motion on one side with motion in the opposite direction on the other (i.e., counterphase movements).

$$\begin{array}{l} \theta_{2,a}=\rho \theta_{1,a} \end{array}$$ (3)

With this approach, a single angular input is used to compute the equilibrium position and velocity of both joints through their kinematic relationship. Any deviation from these equilibrium angles affects the reference component of torque generated on each axis and proportional to the chosen gains. Therefore, assuming that the external equilibrium angle is given in terms of θ_1, *a*_, the resulting reference components for the two joints can be computed as reported in Equation 4.


$$\{\begin{array}{l} \tau_{1,a}=k_{1,a}(\theta_{1,a}-\theta_{1})+d_{1,a}({\dot{\theta }}_{1,a}-{\dot{\theta }}_{1})\\ \tau_{2,a}=k_{2,a}(\rho \theta_{1,a}-\theta_{2})+d_{2,a}(\rho {\dot{\theta }}_{1,a}-{\dot{\theta }}_{2}) \end{array}$$ (4)

##### 2.1.3.2 Bilateral component (τ_*i, b*_)

The bilateral component is used to realize the virtual coupling between the two joints and therefore, by definition, it is set to zero for all applications requiring a decoupled (or monolateral) logic.

Considering all cases of coupled training, instead, the bilateral component is fundamental to promote coordinated motion between the two limbs. Rather than computing the output torque on the basis of an external angular input as in τ_*i, a*_, τ_*i, b*_ produces a restoring visco-elastic field where the equilibrium anchor is defined as the position that each joint should occupy given the current position of the contralateral one. Once again, this kinematic relationship is defined as in Equation 5.


$$\begin{array}{l} \begin{array}{cc} \theta_{1,b}=\theta_{2}/\rho & \theta_{2,b}=\rho \theta_{1} \end{array} \end{array}$$ (5)

As a result, a certain level of torque is produced on each axis proportionally to the deviation from said continuously computed reference, resulting in the mutual coupling of the two joints: when both joints satisfy this relationship simultaneously, no coupling torque is generated, while any deviation from this condition produces a corrective torque that tends to restore consistency. Also in this case, the magnitude of the bilateral torque component can be scaled by tuning the *k*_*b*_ and *d*_*b*_ gains that define the visco-elastic bilateral field.

Additionally, the *bilateral dominance parameter* β∈[−1, +1] is used to control the directional asymmetry of the virtual coupling. At β = −1, the motion of Motor 1 drives Motor 2: deviations of Motor 2 from the position dictated by Motor 1 produce a restoring torque on Motor 2, while Motor 1 remains unaffected. At β = +1, the roles are reversed and Motor 2 drives Motor 1. For intermediate values −1 <β <1, the coupling is bidirectional: both joints influence each other, with a degree of mutual influence that varies continuously from one extreme to the other. For instance, the symmetric case β = 0 produces equal and opposite coupling torques on the two joints. Equation 6 represent the resulting formulas for the three mentioned examples.


$$\{\begin{array}{l} \beta =-1\\ \tau_{1,b}=0\\ \tau_{2,b}=k_{b}(\rho \theta_{1}-\theta_{2})+d_{b}(\rho {\dot{\theta }}_{1}-{\dot{\theta }}_{2}) \end{array}$$

$$\{\begin{array}{l} \beta =1\\ \begin{array}{l} \tau_{1,b}=k_{b}(\theta_{2}/\rho -\theta_{1})+d_{b}({\dot{\theta }}_{2}/\rho -{\dot{\theta }}_{1})\\ \tau_{2,b}=0 \end{array} \end{array}$$

$$\{\begin{array}{l} \beta =0\\ \tau_{1,b}=k_{b}/2(\theta_{2}/\rho -\theta_{1})+d_{b}/2({\dot{\theta }}_{2}/\rho -{\dot{\theta }}_{1})\\ \tau_{2,b}=k_{b}/2(\rho \theta_{1}-\theta_{2})+d_{b}/2(\rho {\dot{\theta }}_{1}-{\dot{\theta }}_{2}) \end{array}$$ (6)

#### 2.1.4 Gamification environment

While providing a dynamic and engaging context to therapy, play is a powerful vehicle for learning and adaptation in pediatric rehabilitation. The design of PhiCube's gamification environment was grounded in empirical evidence on the requirements of children with neuromotor disabilities for accessible technological play, which identified adaptable difficulty and low-threshold physical interaction as key determinants of engagement (36). The effectiveness of this approach has been confirmed in a pilot study evaluating a video game for children with cerebral palsy using PhiCube as the physical controller, which reported high acceptability and satisfaction across a broad spectrum of motor impairment severity (37). Based on these principles, the software compartment of PhiCube has been enriched with a dedicated Graphical User Interface (GUI) that, on top of acting as an intuitive tool for therapists and caregivers in the setting and tuning of the configuration of the device, offers a series of games designed to stimulate various fundamental competences.

**Figure 6:**
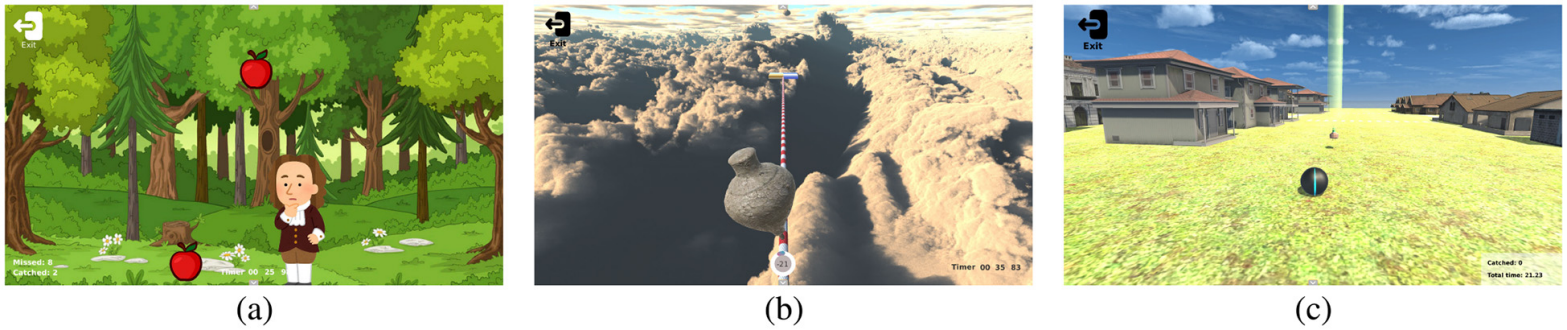
Three examples of therapeutic games provided by PhiCube's GUI that stimulate different motor abilities: **(a)** Newton game; **(b)** Equilibrist game; **(c)** Explorer game.

Of course, a first addressed sphere is movement which represents the only way of interaction between the patient, PhiCube and the virtual environment displayed by the chosen game. Different games have been developed to offer a coherent context to the movement to be trained and to the specific therapeutic goal. For instance, some games aim to stimulate the patient's monolateral ability, some require bilateral symmetrical movements while others work on motor coordination with the two limbs working simultaneously and independently. By matching the patient's residual motor ability, the therapeutic goal of each session, the configuration of the device, the desired gamified activity and its difficulty parameters, it is possible to create highly personalized therapy plans that span across a wide range of conditions. Some examples are depicted in Figure 6. On the left (Figure 6a), the so-called *Newton* game requires the patient to move the character left and right in order to collect the falling apples. One way to play this game is to set PhiCube's central body to the *Vertical* orientation and attaching one *Cycle* handle on the top interface, realizing a monolateral configuration. By rotating the handle in one direction or the other, the patient is able to coherently control the game character to reach the goal. Always with reference to Figure 6, the screenshot in the center (Figure 6b) shows the *Equilibrist* game, where the spinning top needs to be pushed along the rope without falling. This game is perfectly suited for the stimulation of bilateral coupled movements (e.g., *Sagittal* orientation with *FlexiLever* handles installed on each interface), where any asymmetry in the patient's movement on the two sides would cause the spinning top to lean on one side. On the right (Figure 6c), instead, the so-called *Explorer* game requires two different movements on the two sides, one controlling front and back direction and the other commanding left and right movements. Combining these two movements allows the player to move inside a small city to collect randomly spawned objects while training coordination abilities in an engaging way.

**Figure 7:**
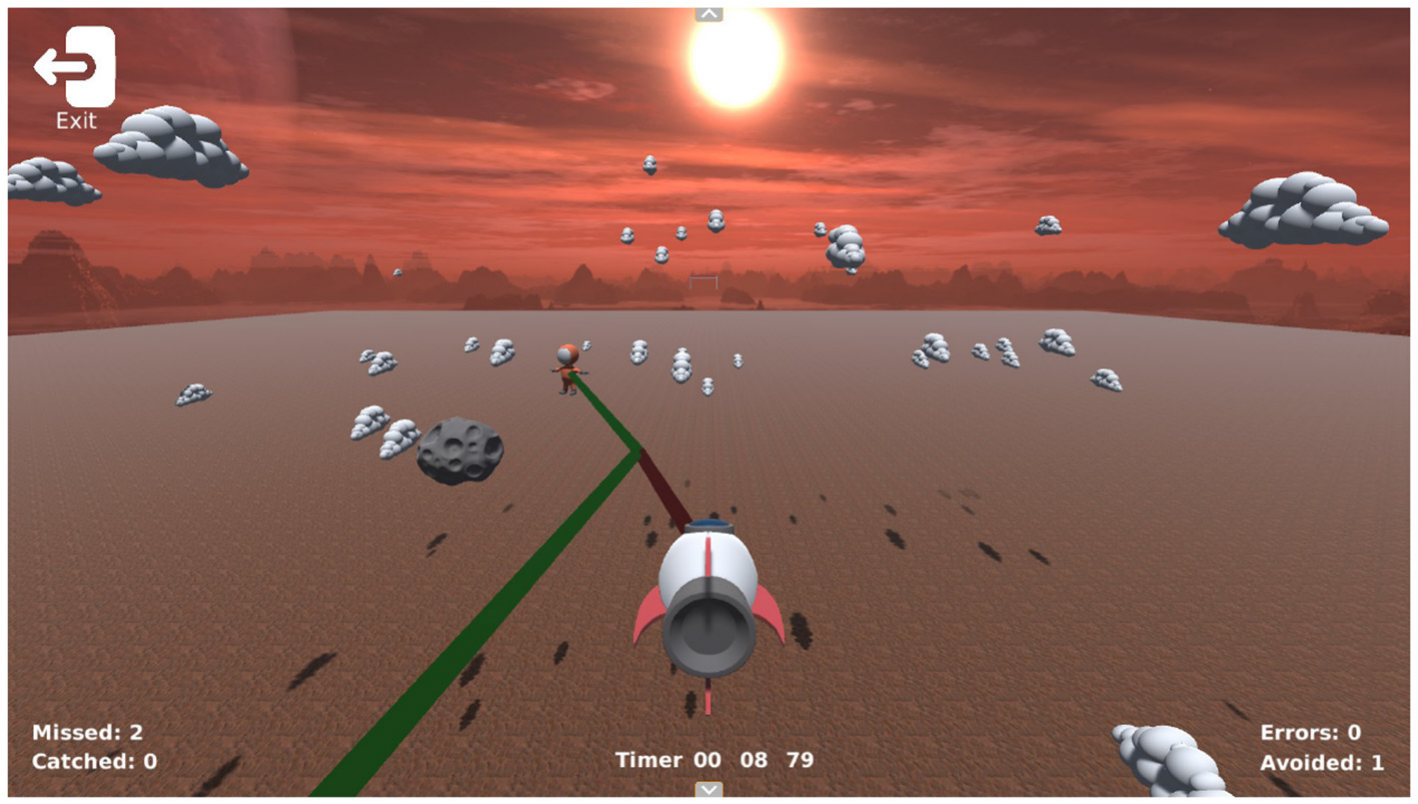
The Shuttle game example provided by PhiCube's GUI integrating features that stimulate cognitive and visual competences as well as generating references for the robotic assistance.

Difficulty, game goal, duration and several other parameters can be tuned to adapt the proposed activity to desired levels. On top of that, since movement is known to be a promoter of learning, it is possible to leverage the patient motor activation to enrich the therapy with the training of other spheres. Therefore, each game offers a number of additional settings that allow to introduce in the proposed activity new elements designed to stimulate secondary competences such as cognitive or visual abilities. For instance, Figure 7 depicts the so-called *Shuttle* game where the goal is to move the starship left and right to save the astronauts lost in space. However, on top of this motor training, the game can be set to also spawn distractor objects in order to stimulate cognitive abilities such as inhibition. Also, the spawning probability of each entity can be tweaked toward the left or right side of the screen in order to address issues of visual neglect that characterize many of the patients with neurodevelopmental disorders.

Always with reference to Figure 7, red and green traces are displayed in front of the player. The green path represents the ideal path to be followed for a perfect game execution while the red path shows how the patient should correct the trajectory to move back to the ideal one if not precisely followed. This feature, together with providing a visual feedback to the patient, is representative of the *reference component* of torque described in Section 2.1.3: the ideal trajectory is mapped in real-time to corresponding equilibrium angle/s (θ_*i, a*_) and sent to the device controller if a certain robotic assistance level needs to be generated to guide the patient.

### 2.2 OpenSim simulation framework

OpenSim is an open-source biomechanical modeling and simulation platform widely used in musculoskeletal research and rehabilitation engineering (38). This software enables the creation, analysis, and visualization of neuromusculoskeletal models, allowing researchers to investigate human movement dynamics, muscle-tendon mechanics, and joint kinematics. OpenSim provides a comprehensive toolkit for inverse kinematics, inverse dynamics, and forward dynamics simulations, making it particularly valuable for analyzing rehabilitation interventions and assistive device interactions with the human musculoskeletal system.

In this study, OpenSim was used for kinematic simulations to evaluate the interaction between PhiCube in its different mechanical configurations and upper-limb biomechanics. The biomechanical modeling approach enabled systematic assessment of how PhiCube's various mechanical configurations elicit distinct patterns of articular motion across the upper extremity kinematic chain. By simulating device-guided movements in different therapeutic setups, we quantified the differential recruitment of joint degrees of freedom (DOFs), providing objective evidence for the device's capacity to target specific movement patterns according to rehabilitation objectives.

**Figure 8:**
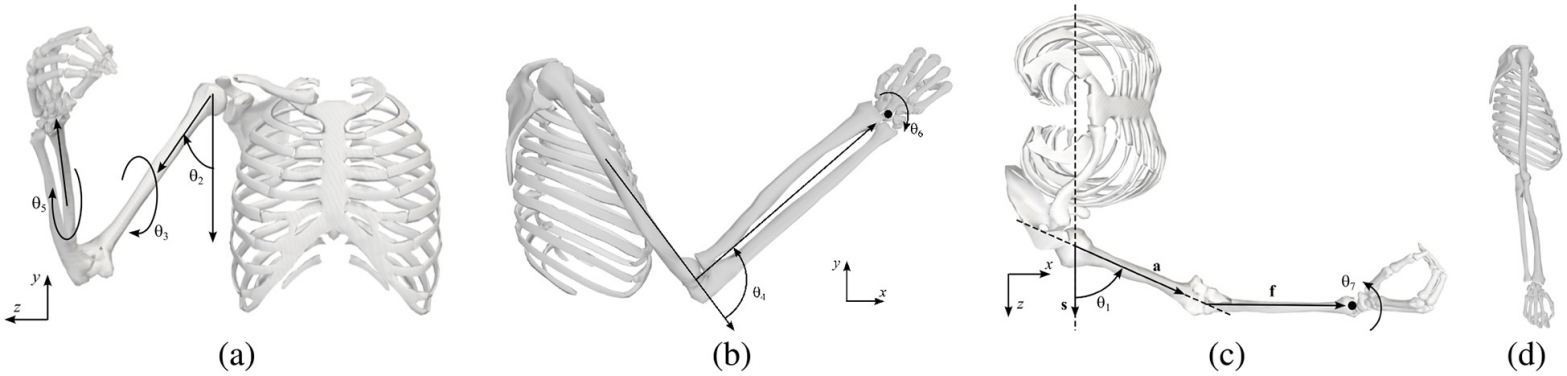
Degrees of freedom of the upper limb kinematic model. θ_1_ Plane of Elevation (PoE), θ_2_ Shoulder Elevation (ShE), θ_3_ Shoulder Rotation (ShR), θ_4_ Elbow Flexion (ElF), θ_5_ Forearm Pronation-Supination (FPS), θ_6_ Wrist Deviation (WrD), and θ_7_ Wrist Flexion (WrF). **(a)** Frontal view. **(b)** Sagittal view. **(c)** Transverse view. On the right, sub-figure **(d)**, the upper-limb configuration in which all DOFs have a value of zero is represented.

The simulations utilized the coordinate system recommended by Wu et al. (39), which has become the standard reference framework in upper extremity biomechanical analysis. Figure 8 illustrates the DOFs of the upper limb kinematic model adopted in this work, which are the same employed in the leveraged musculoskeletal model. The choice of this particular coordinate system ensures consistency and comparability with established literature in shoulder and arm kinematics. Moreover, this kinematic representation allows characterization of the three-dimensional movement capabilities of the upper extremity. The kinematic model does not account for DOFs distal to the wrist, as finger kinematics are highly dependent on individual grasping strategy and play a limited role in the majority of the targeted rehabilitation movements.

For the biomechanical analysis, we adopted the unilateral upper extremity model version 4.1 available at Saul and Murray (40). The computer model was modified from Saul et al. (41) as described by McFarland et al. (42) to include an updated range of motion at the shoulder, ligaments models representing the glenohumeral and coracohumeral ligaments, and updated muscle model (43) with force-length and tendon curves matching the original model's respective curves. The choice of a single-arm model follows directly from the device architecture. The two motorized axes of PhiCube are mechanically independent and share only the common ground of the central body chassis; any inter-limb coordination is realized exclusively at the control level (Section 2.1.3). A fixed-trunk assumption is further adopted as a deliberate methodological choice, to restrict the analysis to the variables determined by the device itself and to exclude subject-specific postural factors—anthropometry, residual postural control, seating arrangement—that are not properties of the device. Under this assumption, the shoulder origins remain stationary and each arm forms an independent closed kinematic chain from the shoulder to the manipulandum; the articular trajectory of each side depends solely on its local motor rotation and on the mounted manipulandum geometry. No degree of freedom is shared between the two sides, and a two-arm simulation would by construction reproduce the same per-side trajectories as two independent single-arm simulations. The fixed-trunk condition is an idealization that may be approximated by a trunk-stabilizing seating arrangement; however, the device does not necessarily require the use of such seating arrangement.

**Figure 9:**
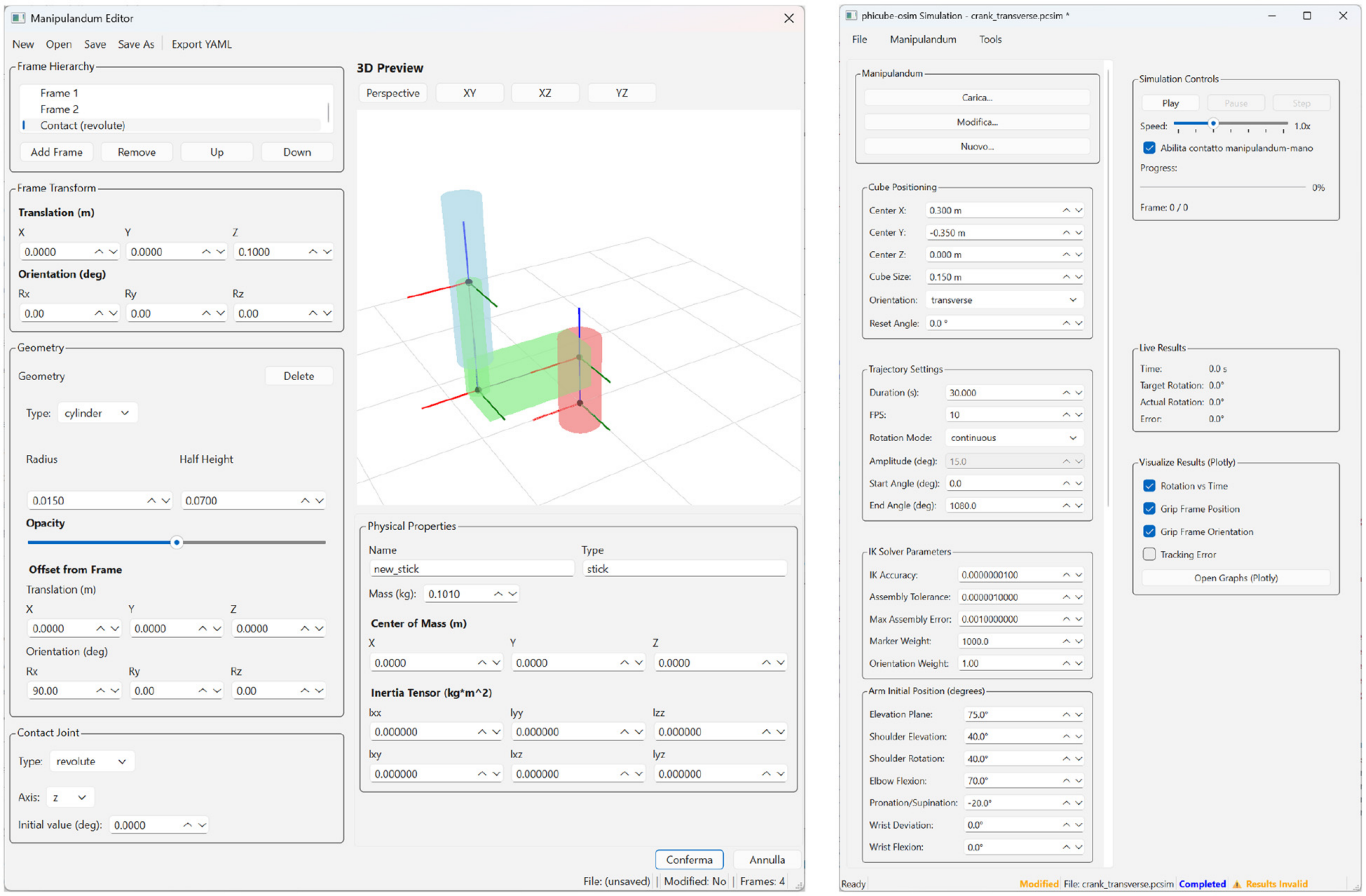
The Python-based OpenSim simulation environment: manipulandum model editor **(left)** and simulation setup interface **(right)**.

To accommodate PhiCube's modular and reconfigurable architecture within the OpenSim simulation environment, a custom Python-based framework was developed utilizing the OpenSim Application Programming Interface (API) (Figure 9). This computational tool addresses the challenge of representing a mechanically flexible rehabilitation device with multiple configuration possibilities within a standardized biomechanical modeling environment.

The framework automatically generates OpenSim representations of PhiCube's different manipulandum configurations by modeling them as an independent rigid body (OpenSim Body object). Each of these bodies is defined with appropriate inertial properties and geometric characteristics corresponding to the physical device components. The program allows systematic variation of manipulandum orientations, attachment positions, and central unit rotational configurations, enabling comprehensive exploration of PhiCube's kinematic workspace across its various mechanical settings.

A critical aspect of the simulation methodology involves the mechanical coupling between the upper extremity model and the PhiCube device representation. This connection is established through OpenSim's WeldConstraint class, which creates rigid kinematic constraints suitable for closed-loop kinematic chain analysis. The WeldConstraint enforces fixed spatial relationships between the hand segment of the musculoskeletal model and the corresponding manipulandum body, effectively creating a closed kinematic chain that combines the human arm with the robotic device.

The Python framework orchestrates the complete simulation workflow—constructing device bodies, establishing rigid coupling constraints, configuring the inverse kinematics solver, and executing the kinematic analysis for prescribed trajectories—enabling systematic investigation of how different PhiCube configurations influence upper limb joint recruitment.

#### 2.2.1 Model scaling

The upper extremity musculoskeletal model was developed based on an adult male reference subject. To evaluate the interaction of subjects with different body dimensions—including children—with the manipulandum device, the model must be scaled to match the anthropometry of each subject.

A uniform scaling approach, in which a single scale factor (e.g., derived from standing height) is applied to all body segments, is inadequate for pediatric populations. In fact, children have body proportions that differ significantly from adults. Applying uniform height-based scaling to a child would therefore overestimate limb segment lengths. For this reason, we adopted a *segment-specific* scaling approach, applying different scale factors to each body segment group based on validated regression equations.

Upper-extremity segment lengths are predicted using multivariate regression Equations 7 and 8 validated by (44):


$$\begin{array}{l} L_{\text{arm}}=0.14˙h+0.28˙a+0.41˙s \end{array}$$ (7)

$$\begin{array}{l} L_{\text{forearm}}=0.12˙h+0.01˙w+0.27˙a \end{array}$$ (8)

where *L*_*arm*_ and *L*_*forearm*_ are the arm and forearm lengths, respectively, *h* is standing height (cm), *w* is body mass (kg), *a* is age (years), and *s* is a sex code (*s* = 0 for male, *s* = 1 for female). The inclusion of age as an independent predictor is critical: it captures the change in body proportions during growth, ensuring that at a given height, a child will have shorter predicted limb lengths than an adult.

To determine the hand scaling factor, the scaling factor proposed by Drillis and Contini (45) was adopted (Equation 9):


$$\begin{array}{l} L_{\text{hand}}=0.108˙h \end{array}$$ (9)

The applicability of this height-based proportionality to pediatric populations is supported by the strong correlation (*r* = 0.91) between hand length and standing height reported by Wen et al. (46) in children aged 5–13 years.

When direct measurements of one or more segment lengths were available, the tool employed allowed for the input of specific anthropometric dimensions.

Geometric scaling was applied using the OpenSim Scale Tool API, which scales each body's dimensions, joint translations, mass distribution, center of mass location, inertia tensor, muscle fiber lengths, tendon slack lengths, and wrapping surface geometries by the corresponding segment factor.

## 3 Results

### 3.1 Kinematic analysis

This section reports results on how different configurations of the PhiCube device, obtained by combining different orientations of the central body and different manipulanda, influence the upper limb's degrees of freedom (DOFs) of movement.

**Figure 10:**
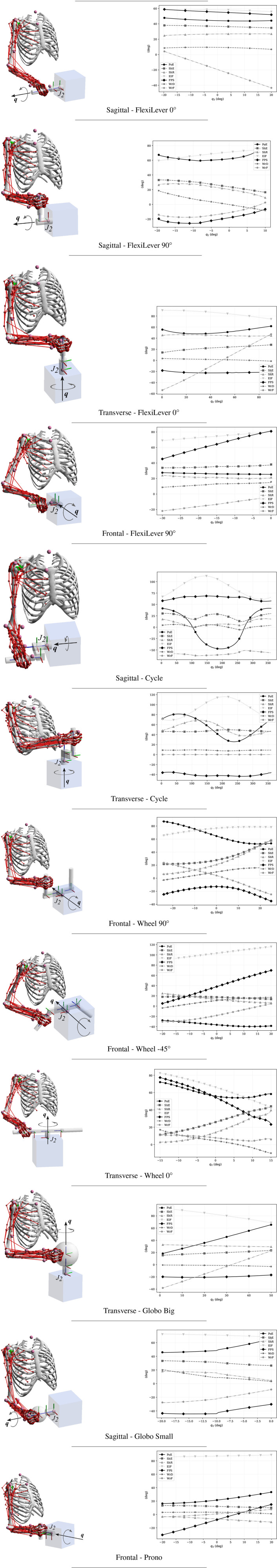
Range-of-motion profiles as a function of PhiCube orientation and the manipulandum used. **(a)** Sagittal - FlexiLever 0°. **(b)** Sagittal - FlexiLever 90°. **(c)** Transverse - FlexiLever 0°. **(d)** Frontal - FlexiLever 90°. **(e)** Sagittal - Cycle. **(f)** Transverse - Cycle. **(g)** Frontal - Wheel 90°. **(h)** Frontal - Wheel -45°. **(i)** Transverse - Wheel 0°. **(j)** Transverse - Globo Big. **(k)** Sagittal - Globo Small. **(l)** Frontal - Prono.

The objective of this analysis is to characterize the contribution of the mechanical reconfigurability of the platform to its therapeutic flexibility, in isolation from the bilateral coupling law addressed in Section 3.2. The range-of-motion profiles reported in Figure 10 express geometric relationships between the rotation angle of each motorized axis and the corresponding articular angles of the arm: they depend on the device mechanical setup, independently of how the motor trajectories are dynamically realized and of any control-level coordination between the two motors.

As described in Section 2.2, the two arm–manipulandum kinematic chains are decoupled within the model, and the recruitment of each limb depends on its local manipulandum–orientation combination. Since PhiCube admits asymmetric setups, each bilateral configuration is identified by the pair of single-side configurations, combining the profiles obtained in this section. The same description trivially extends to intrinsically bilateral accessories, whose construction imposes mechanically symmetric ranges of motion on the two limbs by design, so that the corresponding bilateral profile reduces to a mirror-symmetric pair of single-side profiles.

The analyzed configurations represent a set of relevant combinations obtainable with the handles currently available for PhiCube. They constitute a representative—though not-exhaustive cross-section of the device's reconfiguration space, sufficient to characterize the kinematic opportunity offered by the system across the principal anatomical planes and across the diverse manipulanda available. Future expansions of the manipulanda set will naturally extend this analysis.

Simulation results are represented as joint angle profiles plotted as a function of the motor rotation angle *q*_*i*_ of *J*_*i*_ (with *i* = 2 because the upper limb model is right-handed), rather than as time series. This representation directly exposes the geometric coupling between manipulanda motions and limb kinematics, independently of movement speed or direction. The trajectories can be executed in either direction, providing flexibility in exercise design according to therapeutic goals. Each configuration panel pairs the kinematic profile with a rendering of the scaled musculoskeletal model at the reference posture corresponding to $q_{2}=0^{˙}$ for that specific manipulandum–orientation combination, which varies across configurations depending on the selected manipulandum and central body orientation. The positive rotation direction for *q*_2_ is indicated in each rendering. The plotted joint angle coordinates follow the definitions and sign conventions introduced in Figure 8.

### 3.2 Bilateral coupling controller

This section presents the results of the coupled mode controller analyses. The controller torques (Section 2.1.3)—decomposed into the reference component (τ_*i, a*_), the bilateral component (τ_*i, b*_), and their sum (τ_*i*_)—are computed over a grid of static postural configurations and visualized as contour maps, with the aim of characterizing how the controller parameters shape the torque field and the resulting equilibrium structure. The analyses are quasi-static, therefore the contribution of the damping coefficients are negligible.

The coupled mode controller output is decomposed into two additive contributions for each motor. The reference component (τ_*i, a*_) drives motor *i* toward a fixed equilibrium angle θ_*i, a*_ derived from an external reference, independently of the state of the contralateral side. The bilateral component (τ_*i, b*_) instead generates a torque that depends on the relative angular displacement between the two motors, establishing a mechanical link between the two limbs. The total torque (τ_*i*_ = τ_*i, a*_+τ_*i, b*_) is the net command applied to each motor at any given configuration. All three quantities are represented and analyzed through contour maps defined over the two-dimensional joint-angle space $\left(\theta_{1},\theta_{2}\right)\in {\left[-180^{˙},+180^{˙}\right]}^{2}$, where θ_1_ and θ_2_ denote the angular positions of Motor 1 and Motor 2, respectively. Each point in this space represents a static postural configuration of the two limbs; the contour value at that point indicates the torque that the controller would apply to each motor in that configuration.

To interpret these maps, some considerations are reported here:

- Zero-torque contours as equilibria. A contour value of zero corresponds to a configuration in which the torque applied by the controller exerts no net torque on that motor. In the absence of external loads and in quasi-static conditions, the system tends to evolve toward these zero-torque loci. When both τ_1, *b*_ and τ_2, *b*_ are simultaneously zero, the configuration is a global bilateral equilibrium.
- Torque sign and direction. Positive torque values indicate that the controller pushes the motor in the positive angular direction; negative values indicate the opposite. The gradient of the torque field is therefore a restoring field: the farther the system is from the zero-torque locus, the larger the magnitude of the corrective torque.
- Equilibrium under external loads. When an external torque acts on one or both joints—as occurs during patient–device interaction—the static equilibrium shifts to the configuration at which the controller torque balances the external load. In this case the operating point moves away from the zero-torque contour, and the device controller provides a sustained restoring force proportional to the displacement.
- Coupling vs. independence. If the zero-torque contour of τ_*i, b*_ is a horizontal or vertical line in the (θ_1_, θ_2_) plane, then the equilibrium of motor *i* is independent of the position of the other motor. Conversely, if the zero-torque contour is oblique—passing through both axes—the equilibrium of motor *i* depends on the current position of the contralateral motor, realizing a position-dependent inter-limb coupling.

Each of the following panels presents two side-by-side subplots showing the torques applied by the device to Motor 1 (left) and Motor 2 (right), respectively, allowing the combined action of the controller on both joints to be examined simultaneously. Where two or more subplots within the same figure represent quantities of the same type, a common color scale is adopted to enable direct comparison of magnitudes. To facilitate the interpretation of the figures, both joints share the same value of the the gain controlling the elastic action of the reference torque component (*k*_1, *a*_ = *k*_2, *a*_ = *k*_*a*_).

**Figure 11:**
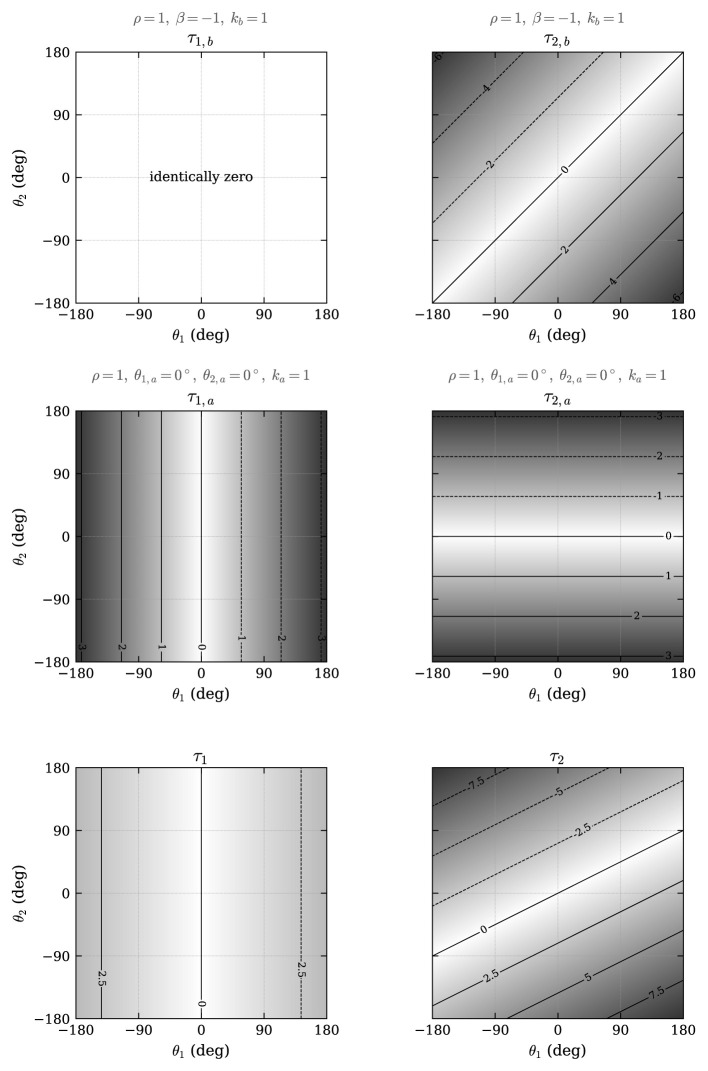
Contour maps of τ_*i, b*_ **(top)**, τ_*i, a*_ **(middle)**, and τ_*i*_ **(bottom)** for Motor 1 (left) and Motor 2 (right), with ρ = 1, β = −1, *k*_*b*_ = 1, $\theta_{1,a}=0^{˙}$, $\theta_{2,a}=0^{˙}$, *k*_1, *a*_ = *k*_2, *a*_ = *k*_*a*_ = 1. τ_1, *b*_ is identically zero. τ_2, *b*_ vanishes along θ_2_ = θ_1_. τ_*i, a*_ acts equally on both joints with no reciprocal influence and vanishes along the $\theta_{i}=0^{˙}$ lines.

Figure 11 (ρ = 1, β = −1) illustrates the maximally asymmetric coupling in which Motor 1 drives Motor 2 with no reciprocal influence. The bilateral reference for Motor 2 is set equal to the current position of Motor 1, so τ_2, *b*_ vanishes along the main diagonal θ_2_ = θ_1_ and increases monotonically in magnitude as the two positions diverge. The torque on Motor 1, τ_1, *b*_, is identically zero across the entire configuration space, confirming that Motor 1 is completely decoupled from Motor 2 under this setting. The diagonal zero-torque contour of τ_2, *b*_ is therefore the locus of equilibrium configurations for Motor 2: regardless of where Motor 1 is positioned, Motor 2 is driven to replicate that position.

**Figure 12:**
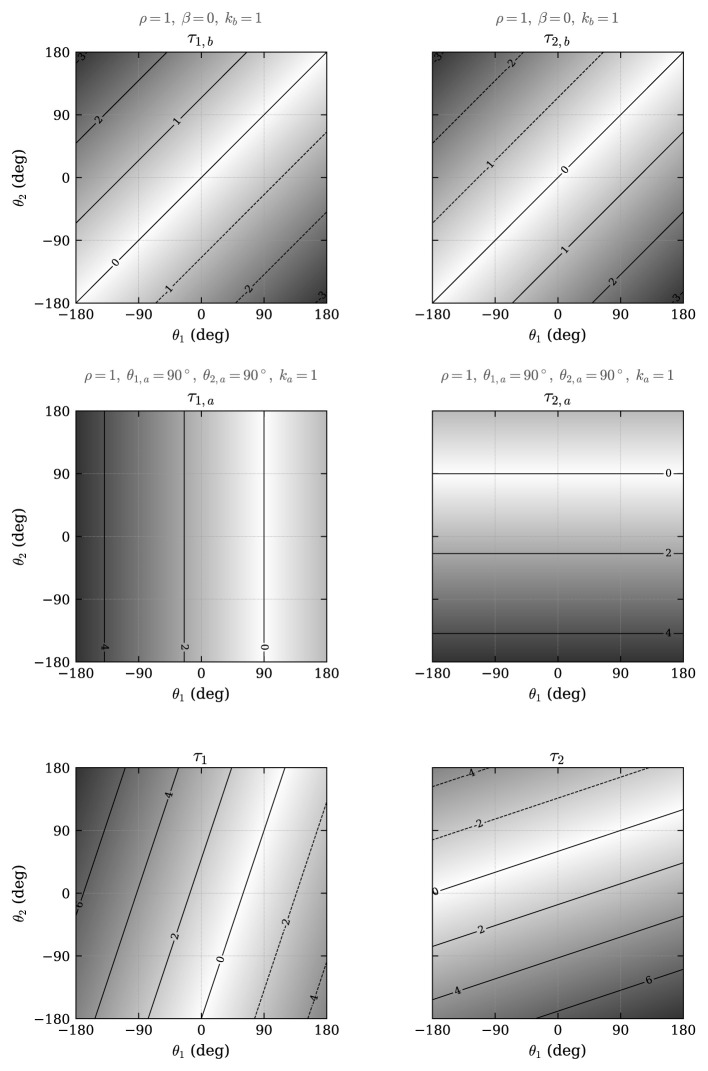
Contour maps of τ_*i, b*_ **(top)**, τ_*i, a*_ **(middle)**, and τ_*i*_ **(bottom)** for Motor 1 (left) and Motor 2 (right), with ρ = 1, β = 0, *k*_*b*_ = 1, $\theta_{1,a}=90^{˙}$, $\theta_{2,a}=90^{˙}$ and *k*_1, *a*_ = *k*_2, *a*_ = *k*_*a*_ = 1. Both τ_*i, a*_ and τ_*i, b*_ act equally on both joints, with the reference components vanishing along $\theta_{i}=90^{˙}$ and the bilateral components vanishing along θ_2_ = θ_1_.

Figure 12 (ρ = 1, β = 0) shows the fully symmetric bilateral coupling. Both τ_1, *b*_ and τ_2, *b*_ are non-zero and depend on both θ_1_ and θ_2_. Each motor receives a torque that drives the system toward the mutual equilibrium located on the main diagonal θ_2_ = θ_1_: the zero-torque contour for both motors coincides with this line. In the absence of external loads on both joints, the bilateral equilibrium is any configuration on the diagonal—that is, any configuration in which the two manipulanda occupy the same angular position. An asymmetric external load applied to one limb will displace the operating point off the diagonal, and the controller will exert a restoring torque on both motors proportional to the inter-limb angular difference.

**Figure 13:**
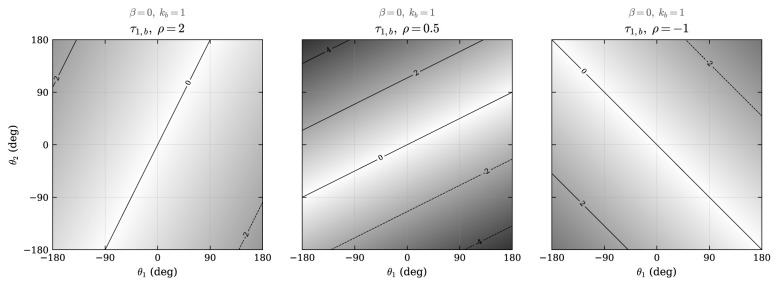
Bilateral component τ_1, *b*_ for ρ = 2, ρ = 0.5 and ρ = −1 **(left to right)**, keeping β = 0, *k*_*b*_ = 1 (common color scale). The zero-torque contour follows θ_2_ = ρθ_1_; the torque gradient magnitude is invariant across all three cases.

Figure 13 presents τ_1, *b*_ for three values of the transmission ratio (ρ = 2, ρ = 0.5, ρ = −1) at fixed β = 0 and *k*_*b*_ = 1, all plotted on a common color scale. The transmission ratio ρ rotates the zero-torque locus in the (θ_1_, θ_2_) plane without altering the magnitude of the restoring torque per unit displacement. For ρ = 2, the equilibrium line is θ_2_ = 2θ_1_: Motor 2 is driven to a position twice as large as that of Motor 1. For ρ = 0.5, the equilibrium is θ_2_ = 0.5θ_1_. For ρ = −1, the zero-torque line becomes θ_2_ = −θ_1_, corresponding to a counter-rotation equilibrium in which the two manipulanda are displaced symmetrically in opposite directions. The common color scale confirms that the gradient of the torque field—and hence the restoring stiffness—is identical across all three cases: ρ selects the target kinematic relationship between the two sides, but *k*_*b*_ alone determines the coupling strength.

**Figure 14:**
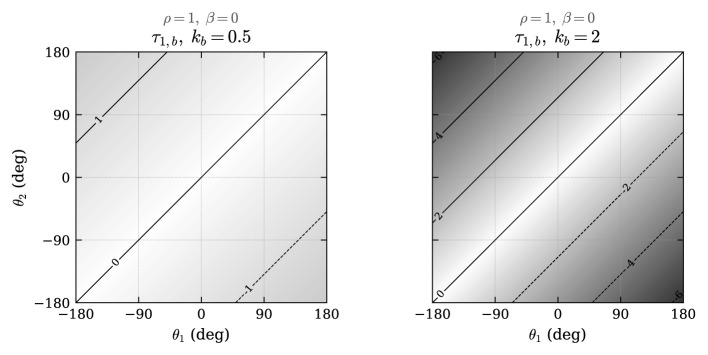
Bilateral component τ_1, *b*_ for *k*_*b*_ = 0.5 and *k*_*b*_ = 2 **(left to right)**, keeping ρ = 1, β = 0 (common color scale). The zero-torque contour is invariant on θ_2_ = θ_1_; the torque gradient scales linearly with *k*_*b*_.

Figure 14 presents τ_1, *b*_ for two values of the bilateral assistance level (*k*_*b*_ = 0.5 and *k*_*b*_ = 2) at fixed ρ = 1 and β = 0, plotted on a common color scale. The zero-torque line remains the diagonal in both cases, confirming that *k*_*b*_ does not alter the equilibrium configuration. What changes is exclusively the gradient of the torque field: for *k*_*b*_ = 2 the torques are exactly four times those for *k*_*b*_ = 0.5, reflecting the linear scaling of τ_*i, b*_ with *k*_*b*_. A higher bilateral assistance level therefore produces a stiffer coupling—a stronger restoring force for any given inter-limb angular discrepancy—without shifting the target relationship between the two limbs.

## 4 Discussion

This section discusses the results presented in Section 3, interpreting them in terms of their biomechanical and clinical implications for upper-limb neuromotor rehabilitation, and highlighting how its modular architecture and bilateral control enable a single platform to address a broad spectrum of therapeutic objectives and patients' needs.

### 4.1 Kinematic reconfigurability and upper limb coverage

The following discussion refers to the results obtained in Section 3.1 through inverse-kinematics simulation, and interprets them in terms of their biomechanical and clinical implications.

The results confirm the core design premise of PhiCube: the combination of central body orientation and manipulandum selection constitutes a systematic instrument for targeting specific DOFs across the seven-DOF shoulder–elbow–forearm chain. This property carries direct clinical relevance for the pediatric population. Children with neuromotor disorders characteristically present with impairments that are not uniformly distributed along the kinematic chain. A platform capable of selectively engaging distinct joint assemblies through rapid mechanical reconfiguration, without requiring separate equipment or prolonged setup, therefore offers a meaningful practical advantage in pediatric clinical settings where session time is limited, therapeutic goals evolve with the child's development, and patient cooperation may be variable.

#### 4.1.1 FlexiLever manipulandum

The results obtained for the FlexiLever manipulandum illustrate how manipulandum inclination angle interacts with central body orientation to shift therapeutic emphasis along the kinematic chain.

##### 4.1.1.1 Sagittal—FlexiLever 0°

In this configuration, wrist flexion–extension emerges as the dominant upper-limb kinematic response, varying almost equally to the manipulandum rotation angle and representing the largest single-DOF excursion across the simulated range. The other DOFs remain comparatively stable. The obtained pattern identifies this setting as a primarily *wrist-targeted* exercise protocol. The capacity to drive wrist flexion–extension in a near-isolated fashion, while the proximal chain is held in an almost stable posture, is of therapeutic relevance for patients with spastic wrist flexor patterns where the clinical objective is to increase dorsiflexion range of motion.

##### 4.1.1.2 Sagittal—FlexiLever 90°

With the manipulanda rotated to 90°, the recruitment pattern changes substantially. Several DOFs of the upper limb are involved in a paired manner. In particular, the shoulder elevation changes progressively coupled with elbow flexion, consistent with a flexion-extension movement of the forearm. These movements are coupled with wrist DOFs. Pronation-supination can be considered as a side-effect of the whole coupling, due to the rigid coupling between the hand and the manipulandum.

This compound pattern kinematically resembles a forward reach-and-release gesture combining glenohumeral elevation (flexion) with elbow extension, a synergy that can be impaired in spastic hemiplegia, where voluntary elbow extension can be compromised. Training this multi-joint coordination pattern, in which the proximal and distal joints move cooperatively in a functionally relevant direction, can be relevant in upper-limb rehabilitation treatments.

##### 4.1.1.3 Transverse—FlexiLever 0°

This configuration primarily engages wrist flexion–extension as the dominant DOF, in a pattern qualitatively analogous to the Sagittal–FlexiLever 0° configuration. The shoulder complex and elbow participate with moderate and relatively stable values, without contributing large excursions across the motor range.

A clinically meaningful distinction from the sagittal configuration lies in the forearm posture: the transverse orientation positions the forearm in a substantially different pronation–supination angle—approximately 90° relative to the sagittal case. This difference is not merely geometric: the functional profile of wrist motion is redefined by forearm orientation, as biomechanical coupling dictates that the force-generating capacity of wrist motors fluctuates according to the degree of pronation (47, 48). Training wrist flexion–extension across different forearm orientations can broaden the motor learning stimulus.

##### 4.1.1.4 Frontal—FlexiLever 90°

This configuration engages different DOFs, with pronation–supination and wrist flexion–extension both contributing substantial excursions across the simulated range. This represents a qualitative shift relative to the two FlexiLever configurations discussed above—Sagittal 0° and Transverse 0°—which each engaged wrist flexion–extension in relative isolation under different forearm orientations. Here, this configuration introduces forearm rotation, producing a compound distal gesture.

This coupling closely replicates the kinematics of operating a door handle—a functional gesture of high relevance in daily living. The ecological validity of this motor pattern, combined with the capacity to practice it in a repetitive, device-guided context with adjustable assistance, makes this configuration suited for task-oriented training.

#### 4.1.2 Cycle manipulandum

The Cycle manipulandum supports continuous rotary motion across more than one central body orientation, engaging multiple DOFs simultaneously in a periodic pattern that does not require reversal of movement direction. This characteristic distinguishes it from lever-type handles, whose oscillatory trajectory implies a direction change at each end of the range. The Cycle manipulandum is well-suited for high-repetition motor training. It should be noted that continuous full rotation is not compulsory: the handle can equally be employed for partial, oscillatory arcs when the patient's range of motion or the therapeutic objective does not require complete cycles, preserving the same DOF recruitment pattern over a reduced angular range.

##### 4.1.2.1 Sagittal–Cycle

The *Sagittal–Cycle* configuration engages the upper limb across a broad multi-DOF pattern throughout the full 360° rotation. Shoulder elevation spans a substantial arc as the primary proximal contributor. The arm elevation angle, although nominally exhibiting a wide excursion in the simulation, should be interpreted with caution: at low elevation angles the shoulder model approaches a kinematic singularity, which produces reduced overall shoulder movements for a large arm elevation angle. Elbow flexion participates with moderate amplitude, providing rhythmic elbow recruitment alongside the shoulder, while wrist flexion shows a smaller but non-negligible excursion.

This multi-DOF periodic engagement closely resembles the arm trajectory required for reciprocal reaching—a gesture that mobilizes the shoulder–elbow synergy central to functional upper-limb use. The cyclic structure of the motion additionally supports the exploitation of rhythm-based motor learning mechanisms, which could facilitate movement acquisition and consolidation in children with neuromotor disorders.

##### 4.1.2.2 Transverse–Cycle

The Transverse–Cycle configuration is characterized by dominant elbow flexion, which spans a substantial arc across the rotation, without however covering the full physiological range of flexion–extension. This is accompanied by significant excursions of arm elevation angle and shoulder rotation, both of which contribute as meaningful secondary components throughout the motion. Shoulder elevation and wrist DOFs participate to a lesser degree.

The co-recruitment of elbow flexion and shoulder rotation in the transverse plane reflects the coupling inherent in horizontal reaching and manipulation tasks, where elbow and glenohumeral rotation act in concert to move the hand across an horizontal plane. Training this synergy in a gravity-compensated plane could reduce the postural demand on the shoulder girdle, making this configuration appropriate for children who do not yet have sufficient proximal strength to perform comparable excursions against gravity, while still engaging a functionally relevant multi-DOF movement pattern.

#### 4.1.3 Wheel manipulandum

The *Wheel* manipulandum emulates the biomechanics of a circular steering gesture, providing a task with high ecological validity in daily-life and recreational contexts. The grip inclination of the Wheel manipulandum can be adjusted across multiple angles, allowing the therapist to tailor the kinematic demand to the patient's available range of motion and to progress the exercise incrementally as articular excursion improves over the course of rehabilitation.

##### 4.1.3.1 Frontal–Wheel 90°

This configuration recruits the shoulder complex across all its principal DOFs: arm elevation angle, shoulder elevation, and shoulder rotation all span substantial arcs throughout the motion. Elbow flexion remains at moderate, relatively stable values.

This glenohumeral engagement with a modest elbow flexion corresponds to the upper-limb kinematics of a symmetric bilateral steering gesture. The shoulder emphasis makes this configuration appropriate for children whose therapeutic objective is glenohumeral strengthening, particularly when combined with an antiphase coupling of the contralateral limb.

##### 4.1.3.2 Frontal–Wheel −45°

Pronation–supination emerges as a relevant DOF, followed by a meaningful contribution from elbow flexion and a non-negligible wrist component. The shoulder complex, by contrast, remains comparatively stable throughout the motion. This configuration is relatively more stable since the grips are located below the rotation axis, unlike the Frontal–Wheel 90° variant where the grip is positioned above it, resulting in an intrinsically unstable configuration.

The reduced shoulder demand makes the −45° variant more suited to children with limited shoulder elevation, for whom the 90° configuration may be mechanically prohibitive. At the same time, the limb recruitment—combining forearm rotation, elbow flexion, and wrist motion—engages a relatively rich distal synergy. The two inclinations therefore address complementary patient profiles within the same manipulandum and orientation, and the possibility of adjusting the grip angle continuously to facilitate the usage and change DOF recruitment.

##### 4.1.3.3 Transverse–Wheel 0°

This configuration engages a broad set of DOFs simultaneously. Wrist DOFs participate to a somewhat lesser degree but remain non-negligible, confirming that the kinematic involvement extends across the full proximal-to-distal chain.

This broad multi-DOF recruitment in the horizontal plane, combined with the antiphase movement of the contralateral limb in bilateral mode, produces a coordination pattern analogous to pushing or sliding objects across a surface. The transverse orientation further reduces gravitational demand on the shoulder girdle, making this configuration accessible to patients with limited proximal strength while still engaging a functionally rich upper-limb synergy.

#### 4.1.4 Globo manipulandum

##### 4.1.4.1 Transverse–Globo Big

This configuration is characterized by a notable coupling between elbow extension and palmar flexion, which co-vary as the dominant kinematic pair throughout the motion. Plane of elevation angle undergoes a considerable excursion; however, given the limited variation in shoulder elevation, this translates into relatively modest displacement of the distal humerus rather than a true large-amplitude glenohumeral movement.

The large-diameter spherical grip is specifically designed for bilateral simultaneous engagement: both hands contact a single interface, promoting symmetric bilateral activation and requiring sustained palmar contact—a grasp format that facilitates passive hand opening in children with spastic flexor patterns who cannot yet voluntarily extend their fingers.

##### 4.1.4.2 Sagittal—Globo small

In the simulated configuration, wrist deviation emerges as a notable contributor, a consequence of the hand resting against the side of the sphere. Unlike Sagittal - FlexiLever 0° whose grip geometry constrains almost hand posture to a single orientation, the spherical shape of Globo allows the patient to approach the manipulandum from multiple directions. Repositioning the hand on the sphere redistributes the contact point, shifting the dominant distal DOF, without any mechanical adjustment of the device. This flexibility also promotes passive maintenance of an open hand posture during interaction. Furthermore, since the two Globo Small handles are mechanically independent—one per motor axis—the bilateral control system can establish any desired coupling relationship between the two limbs.

#### 4.1.5 Prono manipulandum

##### 4.1.5.1 Frontal–Prono

The Prono configuration in frontal orientation provides one of the most kinematically targeted exercises available within the PhiCube system. Pronation–supination emerges as the predominantly varying DOF, while the remaining degrees of freedom remain comparatively stable throughout the motion. This selective engagement of the forearm rotation axis confirms the biomechanical rationale of the handle design.

Forearm pronation–supination deficit can be a functionally significant impairment, affecting the ability to receive objects from others, to orient the hand for grasp-and-release tasks, and to perform bilateral activities requiring a supinated posture.

A kinematically similar recruitment pattern is obtained with the Pinch manipulandum. The latter additionally requires active finger force production for precision grip, engaging intrinsic hand musculature that the employed kinematic model—which does not include finger mobility—cannot represent. The choice between the two manipulanda depends on the patient's level of hand function: the Prono manipulandum is appropriate when the therapeutic focus is forearm rotation in isolation, while the Pinch manipulandum extends the demand to fine motor control of the digits when sufficient finger function is available.

These simulation results should be contextualized within the broader clinical picture. The reported joint angle profiles represent the kinematic response of a scaled musculoskeletal model to device-prescribed trajectories, modeling a scenario in which the limb passively follows the handle motion. In clinical practice, actual joint recruitment may be influenced by the patient's residual voluntary motor function, the degree of spasticity, and the selected assistance–resistance parameters.

### 4.2 Bilateral coupling controller

The contour map analysis presented in Section 3.2 characterizes the torque field generated by the coupled-mode controller across the full two-dimensional joint-angle space. The configurations discussed in the following subsections and illustrated in Figures 11–14 are selected as paradigmatic cases, chosen to isolate the effect of individual parameters on the torque field structure and to illustrate their correspondence to clinically interpretable therapeutic modalities. They do not constitute an exhaustive enumeration of the device's operational space: the continuous and largely independent nature of the parameter set permits a broad continuum of intermediate configurations. The selection of a specific parameter combination for a given patient is therefore a clinical decision, guided by the patient's functional profile, the therapeutic objectives of the session, and the progression of motor recovery over time.

#### 4.2.1 Asymmetric coupling: guided movement for the more-affected limb

The maximally asymmetric configuration (ρ = 1, β = −1), illustrated in Figure 11, implements a *guided movement paradigm* in which the less-affected limb provides a continuous kinematic reference for the more-affected side. Figure 11 presents the decomposition of the controller output into the bilateral component (τ_*i, b*_), the reference component (τ_*i, a*_), and the resulting total torque (τ_*i*_), for a specific combination of parameters chosen as a clinically representative example. This decomposition reveals an architecture in which two distinct assistive actions can coexist on the two limbs, each independently configurable according to the therapeutic requirements of the session.

The bilateral component acting on the less-affected limb (Motor 1) is identically zero throughout the entire configuration space, irrespective of the values assigned to the other parameters: the child moves the less-affected limb freely, without any coupling-induced mechanical interference. The more-affected limb (Motor 2) receives a bilateral corrective torque proportional to its angular deviation from the position simultaneously occupied by Motor 1, translating the child's voluntary movement into a continuously updated therapeutic reference for the impaired upper extremity.

A second, independent action—the assistive reference component τ_*i, a*_—can be optionally activated and calibrated independently on each side through the per-joint gains *k*_1, *a*_ and *k*_2, *a*_, which scale the elastic action exerted on Motor 1 and Motor 2 respectively, with the associated equilibrium angles θ_1, *a*_ and θ_2, *a*_ linked under the coupled logic by the kinematic consistency condition θ_2, *a*_ = ρθ_1, *a*_. In the specific configuration shown in Figure 11 (*k*_1, *a*_ = *k*_2, *a*_, $\theta_{1,a}=\theta_{2,a}=0^{˙}$), the assistive reference component is applied symmetrically on both motors, each one pulled toward its own consistent equilibrium angle: this corresponds to the scenario in which the therapeutic game generates a desired trajectory θ_1, *a*_(*t*) on the less-affected side and the contralateral target θ_2, *a*_(*t*) = ρθ_1, *a*_(*t*) is derived through the consistency condition, and the device provides calibrated guidance on both sides along their respective consistent references, while the asymmetric bilateral action continues to drive Motor 2 toward the position of Motor 1. The contour maps of the assistive reference component in Figure 11 show strictly vertical iso-torque lines for τ_1, *a*_ and strictly horizontal iso-torque lines for τ_2, *a*_, confirming that it depends exclusively on the position of its own motor and is entirely independent of the state of the contralateral one. This orthogonality is an architectural property of the control system: it ensures that the assistive reference component does not introduce any inter-limb coupling beyond the one explicitly imposed by the bilateral component.

The total torque field shown in the bottom row of Figure 11 reflects this separation of roles: Motor 1 receives only the reference contribution (since the bilateral component is identically zero on this side), while Motor 2 receives the superposition of the bilateral and reference contributions. It is important to emphasize that this specific distribution follows from the chosen values of *k*_1, *a*_ and *k*_2, *a*_, and is not a fixed property of the asymmetric coupling mode. Setting *k*_2, *a*_ = 0 recovers the pure guided-movement paradigm in which the more-affected limb is driven exclusively by the bilateral coupling, while the entire reference action is concentrated on the less-affected side. Conversely, activating *k*_2, *a*_ alongside *k*_1, *a*_ superimposes a direct reference action also on Motor 2, complementing the bilateral guidance when this alone is insufficient to keep the more-affected limb on the desired trajectory. Because the two gains are tuned independently, a continuous range of configurations is available, from a reference action restricted to the less-affected limb to a more balanced distribution across both sides, with the kinematic consistency condition θ_2, *a*_ = ρθ_1, *a*_ ensuring that the reference and bilateral targets remain aligned regardless of the chosen distribution.

This configurability gives rise to a coupled assistive architecture when both components are active: the game trajectory guides Motor 1 along the desired path through the reference component, and the position of Motor 1—more accurately maintained along that path as *k*_1, *a*_ increases—in turn serves as the reference for the bilateral coupling that drives Motor 2. This is not a fixed mechanical property but a therapeutic modality that the clinician can selectively activate and tune. For a child whose less-affected limb already executes the game trajectory reliably under voluntary control, the reference component can be disabled entirely by setting *k*_1, *a*_ = *k*_2, *a*_ = 0, preserving the child's active role without unnecessary mechanical intervention. Conversely, on days when motor performance is reduced—due to fatigue, spasticity fluctuations, or attentional difficulties—activating the reference component at a moderate gain provides just enough trajectory support to maintain task coherence without removing the child's contribution to the movement.

#### 4.2.2 Symmetric coupling: bilateral symmetrical movement training

The symmetric bilateral coupling (ρ = 1, β = 0), shown in Figure 12, generates equal and opposite corrective torques on both limbs whenever their angular positions diverge from the shared equilibrium θ_2_ = θ_1_. This paradigm is particularly suited to children with bilateral upper limb involvement—including bilateral spastic cerebral palsy and developmental coordination disorder—in whom an important therapeutic goal is the consolidation of coordinated bilateral patterns. Any tendency for one limb to lead or lag behind the other is continuously counteracted by a restoring torque on both joints, providing real-time haptic reinforcement of the coordinated pattern. The optional reference component τ_*i, a*_, driven in real time by the game trajectory, allows to modulate task difficulty without altering the coupling structure. The convergence of this haptic bilateral signal with the visual error feedback provided by games (e.g., the *Equilibrist*) is consistent with multimodal feedback principles known to accelerate motor learning in children.

A noteworthy property of the β = 0 configuration is that the symmetric torque distribution also gives rise to an haptic feedback on the less-affected limb. When the more-affected limb deviates from the shared trajectory, the less-affected limb simultaneously receives a resistive torque proportional to that deviation: the child perceives the bilateral deficit directly through the less-affected hand. This embodied error signal may be clinically exploited in selected hemiplegia presentations. The clinician should however be aware that this benefit comes at the cost of mechanically constraining the less-affected limb, which constitutes a deliberate departure from the pure guided movement paradigm (β = −1) discussed above: the choice between the two configurations reflects a clinical judgement about the relative priority of unconstrained voluntary movement versus augmented haptic awareness at a given stage of rehabilitation.

#### 4.2.3 Transmission ratio: selecting the target kinematic relationship

The parametric sweep of the transmission ratio ρ presented in Figure 13 reveals that this parameter acts exclusively as a selector of the *target kinematic relationship* between the two limbs, without altering the mechanical stiffness of the coupling. By adjusting ρ, the clinician sets the desired angular ratio between the two sides, allowing the device to accommodate functional asymmetries in which the two limbs operate over different ranges of motion. This is particularly relevant in patients where a direct one-to-one correspondence between the excursions of the two limbs is neither achievable nor therapeutically appropriate—such as in the presence of marked inter-limb asymmetry due to spasticity, post-surgical limitations, or developmental imbalance—enabling a graded progression toward symmetric bilateral coordination as motor capacity improves. The counter-phase case ρ = −1 establishes a reciprocal anti-symmetric coupling—with the zero-torque locus along θ_2_ = −θ_1_—directly suited to alternating bilateral exercises, where the therapeutic objective is the coordination of out-of-phase rather than synchronous limb movements.

In Figure 13, the bilateral assistance level is held constant (*k*_*b*_ = 1) across all three cases to isolate the sole effect of ρ on the torque field structure; the independent role of *k*_*b*_ in scaling the coupling intensity is examined separately in Figure 14.

#### 4.2.4 Bilateral assistance level: progressive intensity modulation

Figure 14 isolates the effect of the bilateral assistance level *k*_*b*_, demonstrating that this parameter operates as a pure gain on the coupling torque field: the zero-torque contour remains invariant at θ_2_ = θ_1_ for both *k*_*b*_ = 0.5 and *k*_*b*_ = 2, while the torque magnitude scales linearly. This linear relationship underpins the progressive intensity modulation required across the wide spectrum of functional levels encountered in pediatric neuromotor rehabilitation. The clinical significance of this property lies in its direct correspondence to the *assist-as-needed* principle, widely recognized as a key determinant of effective motor rehabilitation: the intensity of mechanical guidance should be continuously calibrated to the patient's residual capabilities, providing just enough support to enable task execution without substituting the child's voluntary effort (49). By modulating *k*_*b*_, the system can navigate a continuous spectrum from a firm coupling—indicated when voluntary motor control is severely limited and the more-affected limb requires strong bilateral guidance to maintain consistency with the other side—to a progressively more permissive regime that tolerates increasing inter-limb discrepancy as the child's motor function improves, gradually reducing mechanical substitution and restoring freedom of movement to the recovering limb.

#### 4.2.5 Flexibility of the control architecture

The parametric analysis presented across Figures 11–14 demonstrates that the coupled-mode controller provides a broad and continuously adjustable space of bilateral interaction modalities. The independent action of its constituent parameters enables a wide range of therapeutic configurations to be realized through the same underlying control architecture, from strict bilateral guidance to permissive coupling, from symmetric to asymmetric inter-limb relationships, from synchronous to reciprocal movement patterns. This flexibility is a key translational property of PhiCube: the device can be configured to match the functional profile of a given patient at a given stage of rehabilitation, and progressively reconfigured as motor capacity evolves, without requiring hardware changes or modifications to the therapeutic setup. This adaptability is particularly relevant in pediatric neuromotor rehabilitation, where the spectrum of clinical presentations and the rate of motor development vary substantially across patients and across time within the same patient.

## 5 Conclusions and future works

This paper presented PhiCube, a modular robotic platform for bilateral upper-limb neuromotor rehabilitation, introducing its mechanical concept, kinematic model, bilateral control architecture, and gamification environment. The kinematic simulations confirmed that the combination of central body orientation and manipulanda selection constitutes an instrument for targeting distinct articular assemblies across the upper limb chain, while the analysis of the controller demonstrated the flexibility and tuneable nature of the bilateral interaction parameters.

PhiCube is currently undergoing clinical validation through a multicentre study involving leading Italian pediatric rehabilitation centers, conducted within the Fit4MedRob research programme, with the aim of establishing evidence-based protocols for children with cerebral palsy and developmental coordination disorder (ClinicalTrials.gov: NCT07092436). Additionally, the obtained results will likely provide more information related to how neuromotor disorders impact the provided kinematic analysis which would allow future modeling of functional impairments to further refine PhiCube design and functionalities.

A GDPR-compliant data collection system is currently operational in its prototypal form, together with a first set of integrated assessment activities enabling the computation of quantitative performance indices. Both the data infrastructure and the assessment protocol will be progressively expanded to support broader longitudinal studies and systematic clinical evaluations across diverse patient populations and rehabilitation settings.

Several directions are planned for future development. Adaptive algorithms will automate the modulation of coupling and assistance parameters in closed loop, operationalizing the assist-as-needed principle without requiring manual therapist adjustment between repetitions. The handle set and the gamification library will be progressively expanded to broaden the range of addressable motor patterns and to incorporate evaluative protocols for systematic longitudinal monitoring of patient progress.

As an end-effector device, PhiCube does not mechanically constrain the proximal upper limb segments, affording rapid setup and broad anthropometric adaptability. The subject's position relative to the device can be deliberately adjusted to modulate the functional demand on specific joint assemblies. However, the absence of proximal constraints leaves compensatory strategies—such as trunk rotation or shoulder elevation—undetected from end-effector kinematics alone. To address this, a markerless skeletal tracking module is under development to monitor patient posture and detect compensatory movements in real time during device interaction.

Future investigations will extend the OpenSim-based analysis into the dynamic domain. The present study is intentionally focused on the kinematic level of description, since the mapping between device configuration and the upper-limb range of motion is geometric, and depends on the active mechanical configuration; this allows the mechanical reconfigurability of PhiCube to be characterized in isolation from the bilateral control architecture, which is examined separately in Section 3.2. From this perspective, the kinematic analysis presented here is equivalent to operating the two motors under position control. A coupled kinematic–dynamic characterization, in contrast, would depend jointly on the active configuration, on the parameters of the bilateral control law, and on the residual neuromuscular profile of the individual subject, and is therefore meaningful only when grounded on subject-specific experimental data. Dedicated future studies are planned to integrate electromyographic recordings on the target patient population, coupling the controller torque field of Section 3.2 with subject-specific musculoskeletal modeling and validating the analysis against measured kinematics, kinetics, and EMG signals.

A functional electrical stimulation system is also envisioned as a future extension of PhiCube, operating in concert with the motor actuation to reinforce neuromuscular recruitment during rehabilitation exercises. In this perspective, ongoing activities are devoted to the analysis of muscle synergies associated with the different device configurations: characterizing how each handle–orientation combination recruits the underlying coordination structures of the upper limb will provide the basis for synergy-informed stimulation patterns adaptively modulated according to the active configuration and the movement being trained.

Beyond cerebral palsy and developmental coordination disorder, PhiCube's reconfigurable architecture and bilateral gamified paradigms may extend to further pediatric neuromotor conditions, including rare diseases for which rehabilitation technology remains largely unavailable. In Rett syndrome, structured purposeful motor engagement may reduce hand stereotypes while simultaneously addressing attentional deficits. In Friedreich's ataxia, bilateral coordination training with augmented visual feedback may provide a compensatory strategy for proprioceptive loss. In pediatric Charcot–Marie–Tooth disease, characterized by progressive distal upper limb weakness and prominent fatigue, self-paced gamified exercises with augmented visual feedback may support motor coordination while accounting for the neuromuscular fatigue that characterizes the disease course. Taken together, these prospective extensions reflect the broader ambition of PhiCube: to provide a single, adaptable platform capable of addressing the heterogeneous spectrum of pediatric neuromotor rehabilitation needs, from the most prevalent conditions to rarer and underserved clinical populations.

## Data Availability

The raw data supporting the conclusions of this article will be made available by the authors, without undue reservation.
